# Circadian clock-controlled gene expression in co-cultured, mat-forming cyanobacteria

**DOI:** 10.1038/s41598-020-69294-3

**Published:** 2020-08-24

**Authors:** Christine Hörnlein, Veronique Confurius-Guns, Michele Grego, Lucas J. Stal, Henk Bolhuis

**Affiliations:** 1 Department of Marine Microbiology and Biogeochemistry, Royal Netherlands Institute for Sea Research, and Utrecht University, Den Burg, The Netherlands; 2 Department of Fresh Water and Marine Ecology, Institute for Biodiversity and Ecosystem Dynamics, University of Amsterdam, Amsterdam, The Netherlands

**Keywords:** Biofilms, Microbial communities, Microbial ecology, Microbiology, Bacterial transcription

## Abstract

Natural coastal microbial mat communities are multi-species assemblages that experience fluctuating environmental conditions and are shaped by resource competition as well as by cooperation. Laboratory studies rarely address the natural complexity of microbial communities but are usually limited to homogeneous mono-cultures of key species grown in liquid media. The mat-forming filamentous cyanobacteria *Lyngbya aestuarii* and *Coleofasciculus chthonoplastes* were cultured under different conditions to investigate the expression of circadian clock genes and genes that are under their control. The cyanobacteria were grown in liquid medium or on a solid substrate (glass beads) as mono- or as co-cultures under a light–dark regime and subsequently transferred to continuous light. TaqMan-probe based qPCR assays were used to quantify the expression of the circadian clock genes *kaiA*, *kaiB,* and *kaiC*, and of four genes that are under control of the circadian clock: *psbA*, *nifH, ftsZ*, and *prx*. Expression of *kaiABC* was influenced by co-culturing the cyanobacteria and whether grown in liquid media or on a solid substrate. Free-running (i.e. under continuous light) expression cycle of the circadian clock genes was observed in *L. aestuarii* but not in *C. chthonoplastes*. In the former organism, maximum expression of *psbA* and *nifH* occurred temporally separated and independent of the light regime, although the peak shifted in time when the culture was transferred to continuous illumination. Although functionally similar, both species of cyanobacteria displayed different 24-h transcriptional patterns in response to the experimental treatments, suggesting that their circadian clocks have adapted to different life strategies adopted by these mat-forming cyanobacteria.

## Introduction

The study of the ecology and evolution of natural microbial assemblages is often hindered by the complexity of these systems as well as by the fluctuations of environmental conditions. Laboratory experiments avoid these drawbacks and their outcomes are sometimes extrapolated in an attempt to understand the ecology of the microbial community in nature. Such laboratory experiments are usually carried out using pure cultures growing under well-defined conditions and high nutrient concentrations and under constant temperature and light. However, the results of these experiments often do not allow to predict the natural behaviour of the microorganisms. The emerging field of synthetic microbial ecology tackles these shortcomings by combining a lower complexity with mimicking conditions of natural ecosystems. This holds the promise of a better understanding of the ecology and evolution of microorganisms in natural ecosystems^1,2^. Currently, applications of synthetic microbial ecology address processes such as recycling of waste products^3^, industrial fermentation^4^, dairy industry^5^, production of chemical compounds^6^, and improvement of human health through fecal transplantation^7^.

Coastal microbial mats are amongst the most complex and diverse ecosystems^8^ and are exposed to microscale gradients (among others light, oxygen, salinity, and sulfide) fluctuating with the day-night and/or tidal cycles^9–11^. The dominant primary producers in these mats are cyanobacteria that form complex assemblages and maintain tight interactions with microorganisms belonging to different functional groups^9^. Cyanobacteria possess a circadian clock, a well-characterized molecular mechanism that produces approximately 24-h rhythms in gene expression and protein activity^12^. True circadian rhythms persist even under continuous illumination (free-run), and are able to shift their phases (timing of highest and lowest expression) according to environmental variations (entrainment), while their period is more or less insensitive to temperature (temperature compensated)^13–15^. The endogenous rhythmicity enables cyanobacteria to anticipate and react to daily environmental changes thereby enhancing their fitness^16,17^. The core genes involved in the cyanobacterial circadian clock are *kaiA*, *kaiB* and *kaiC*. They encode proteins that generate circadian rhythms of KaiC phosphorylation. The phosphorylation state of KaiC is a key regulator of the transcription-translation machinery of the cell. Overexpression of KaiA increases KaiC autophosphorylation while excess of KaiB leads to KaiC dephosphorylation. Synchronization to the environment is regulated by KaiA and CikA through the indirect sensing of light. The bacteriophytochrome-like circadian input kinase CikA plays an important part in the timing of the circadian period by sensing changes in light intensity through the redox state of the plastoquinone pool^18^ and it also acts as a phosphatase that is regulated by the KaiB-KaiC protein complex^19^. Entrainment of the cyanobacterial circadian clock happens also through photosynthetic activity that produces ATP, which is—amongst others—a fundamental requirement for the phosphorylation of KaiC and the production of circadian rhythmicity^20,21^.

The nature of the cyanobacterial circadian clock has been vastly studied in planktonic mono-cultures of the unicellular cyanobacterium *Synechococcus elongatus* PCC7942^22^. The attractiveness to study this model strain is its small genome size, simple cellular structure, accessibility to genetic modification and comprehensive standardization of high-throughput assays. However, little is known about how circadian clock-controlled gene expression is regulated in complex communities and whether the environmental complexity and presence of other species is of influence.

A potential external source of influence on rhythmic gene expression is the complexity of the environment, as was reported for *Staphylococcus aureus* and *Vibrio cholerae* in which gene regulation and metabolic activity depended on whether these bacteria were grown as a homogenized liquid culture or biofilm-grown^23,24^.

An additional source of influence on gene expression might be presented by another, independent circadian oscillator which is linked to the ubiquitous antioxidant enzyme peroxiredoxin. Peroxiredoxin follows a 24-h oxidation–reduction cycle and is highly conserved in all domains of life^25^, however, the underlying mechanisms are not well understood.

To investigate whether growth condition and/or species interaction influences circadian gene expression patterns, co-cultures of two filamentous, non-heterocystous cyanobacteria *Lyngbya aestuarii* and *Coleofasciculus chthonoplastes* were subjected to quantitative PCR-based gene expression analysis. This technique was chosen over metatranscriptomics due to the large number of variables and replicates tested that add up to 648 samples to be analysed. In addition, alternative techniques like the use of reporter genes are not yet available for the two cyanobacterial species. aTargeted genes were the circadian clock controlling genes *kaiABC*, and the clock dependent genes *nifH* (encoding the nitrogenase iron protein), *psbA* (encoding photosystem II protein D1), *ftsZ* (encoding a cell division protein) and *prx* (encoding the peroxiredoxin protein). The quantified expression was normalized against the expression of two housekeeping genes, *rnpA* (ribonuclease P protein) and *ppc* (phosphoenolpyruvate carboxylase). The species were grown in either liquid media or biofilm-grown on glass beads and as mono- or co-cultures. Furthermore, after a standard light–dark regime, the cultures were exposed to continuous light to study the free-running circadian clock.

## Results

### Total RNA and qPCR statistics

The extracted total RNA was of good quality (average RIN: 7.7 ± 1) and yielded higher concentrations from *C. chthonoplastes* than from *L. aestuarii* (Table 1). Co-cultures yielded less total RNA than mono-cultures. Similarly, biofilm-grown cultures yielded less total RNA when compared to liquid cultures (Table 1).

**Table 1:** Average quantity (ng/µl) and standard deviations (SD) and the average quality (RIN) of total RNA extracted from *L. aestuarii* (LA) and *C. chthonoplastes* (CC).

Treatment	Species	ng/µl (± SD)	RIN
ML	LA	24 (± 19)	9
	CC	33 (± 25)	7
CL	LA/CC	22 (± 16)	8
MB	LA	15 (± 18)	9
	CC	10 (± 10)	7
CB	LA/CC	16 (± 13)	6

Average quantities and qualities are given for each treatment (L = liquid, B = immobilized, M = mono-culture, C = co-culture).

The qPCR-derived Ct values of the biological replicates of the targeted circadian clock gene cluster *kaiABC*, nitrogenase gene *nifH*, cell division protein gene *ftsZ*, photosynthesis D1 protein gene *psbA*, peroxiredoxin gene *prx* and two commonly used cyanobacterial housekeeping genes, *rnpA* and *ppc* ranged from 23 to 39 in *L. aestuarii* and from 21 to 38 in *C. chthonoplastes*. The average *r*^2^ and amplification efficiencies of the qPCR assays were 0.95 and 100% in *L. aestuarii* and 0.92 and 92% in *C. chthonoplastes* (Table 2)*.* Regardless the taxon and treatment, the highest absolute transcription was observed for the photosystem II protein D1 coding gene *psbA* with on average 153,425 gene-copies/µl in *L. aestuarii* and 78,592 gene-copies/µl in *C. chthonoplastes*.

**Table 2:** RT-qPCR primer (forward (F), reverse (R)) and TaqMan-probe (P) information for *C. chthonoplastes* and *L. aestuarii*.

Species	Gene	Function	Primer/probe	Sequence (5′- > 3′)	Amplicon size (bp)	Reporter	Amplification efficiency	*r*^*2*^
*C. chthonoplastes*	*cikA*	Circadian input kinase A	2816P	CGGGCAGTGATAATACCCAATCCTGTGGC	123	FAM	0.89	0.95
			2796F	GTAGCGGTTGAGTCACCCAA				
			2918R	AGTCGGACTTTATGGCGCAA				
	*ftsZ*	Filamenting temperature-sensitive mutant Z, cell division protein	984P	ACCAAAACTCGATCCCAGCCACTTCACT	126	FAM	0.90	0.98
			936F	GTTTAGCGGCGGAGTTTTGG				
			1061R	TTGTTGGTGTTGGTGGTGGA				
	*kaiA*	Circadian clock protein A	330P	GGAGGTGCATTATTTGTTTCACGCCGCT	106	FAM	0.80	0.94
			310F	ACCGTTCGTTGTACTCCAGG				
			415R	TTGCCTGTTCCAGAGCATGT				
	*kaiB*	Circadian clock protein B	159P	TGCCGAGGAAGATAAAATTTTGGCGACGC	104	HEX	0.84	0.95
			113F	GGGTTTACGCCCTGAAAGTG				
			216R	CGGAGGGGGTAAAACTTTCG				
	*kaiC*	Circadian clock protein C	539P	TGATGACGACGGAGCGCGTGGA	135	CY5	1.07	0.89
			517F	AAGCAAATTGGCGTGACGAC				
			651R	TCGCCGTTCTCCTTCCAAAA				
	*nifH*	Nitrogenase iron protein	614P	TCGTCGAGATCAACGTCGTCGCCTT	133	HEX	1.22	0.87
			578F	ACGCAGAGGGTTTTGCCATA				
			710R	CCAAAGCAGATTCTACGCGC				
	*psbA*	Photosystem II protein D1	458P	GCACCGCCGAATACGCCAGCC	109	CY5	0.92	0.90
			399F	CGGTGGTTTCACGTACCAGA				
			507R	CTGATGCACCCCTTCCACAT				
	rnpA*	Protein subunit of ribonuclease P	139P	GCGCAAAGCCGCTCGTATCTGACG	134	CY5,5	0.85	0.94
			99F	CTATGACATGCCAACCGGGT				
			232R	CCCCACTCGCATCGGTATTT				
	ppC*	Phosphoenolpyruvate carboxylase (PEPC)	2573R	ACCCAAGGAATTGCCCGTAG	144	ROX	0.89	0.94
			2513P	CCCGTCCCACTCGTCGCGGT				
			2424F	TCGGGGGTTTGTGTATGAGC				
		*Prx *Peroxiredoxin	76P	AGGGCAAACTTCGTCTGGATGCGCT	108	HEX	0.72	0.93
			24F	TGGACTTAACGGGATCGGGA				
			131R	TGCGGACTCTGAAAGCGATT				
*L. aestuarii*	*cikA*	Circadian input kinase A	2323P	TCCCCCGACTTTGGCGATGCGT	101	FAM	1.29	0.9
			2300F	TGATCAATCAAATCCCATGACCA				
			2400R	CAAAAACAAGCCGAAGAAGAACT				
	*ftsZ*	Filamenting temperature-sensitive mutant Z, cell division protein	267P	TGTGGGCGGCAGTGGGGGAA	150	FAM	1.02	0.97
			240F	TAACGCAGCGAAGATTAAAGTCA				
			389R	AAACGTTTCAGTGCTTTTGACTG				
	*kaiA*	Circadian clock protein A	290P	TCTTGGCGTTCGGCTGGAGGCA	119	FAM	1.00	0.98
			220F	AAGGGCTTTATCTCCGGAAAAAT				
			338R	GCAGTTCTAAAAATTTTCTGCGGA				
	*kaiB*	Circadian clock protein B	152P	TCGGCGAGTTGAGGATTTTTCAGAACGTCA	145	HEX	1.07	0.97
			116F	TTAGAAAGGGTCGGTGTGGC				
			260R	CGAACTCTGTACGAGCGCTA				
	*kaiC*	Circadian clock protein C	1081P	CCGTCTAACCACGCCTGCGGC	140	CY5	0.98	0.99
			989F	CGAGCAACCGGACCATATTC				
			1128R	GTGACTGCGGTTTTCCAACA				
	*nifH*	Nitrogenase iron protein	187P	GCGATATTCTGCCGATTGTTCGTGGTCG	147	HEX	0.92	0.95
			127F	GTTGTCAATCGGTGTCGGGA				
			273R	CGCTCCAAGCAGGTACAAGA				
	*psbA*	Photosystem II protein D1	86P	ACATCGGCTGGTTCGGCGTGTTG	112	CY5	1.01	0.90
			50F	TCTGCAACTGGGTAACGTCC				
			161R	GCGACGAAGGCTACGATGTA				0.95
	rnpA*	Protein subunit of ribonuclease P	237P	CGATCCGGGTCGCTCGCTGC	131	CY5,5	1.05	
			199F	CACAGCGCGTTTGCTAACTT				
			329R	CTCACTTAACGCTGAGGGCA				
	ppc*	Phosphoenolpyruvate carboxylase (PEPC)	2905P	GCCAGTGCGCCCGGAGTGATT	118	ROX	1.11	0.91
			2882F	AGCGTTTACGTCAGCATGGA				
			2999R	CGCATTCCAGCAGCAATACC				
	*prx *	Peroxiredoxin	55P	ACGCTGGGTATAATCCGCTTTGACAAACCC	106	HEX	1.02	
								0.98
			28F	TGTAGCGAGAATTTCTTCTGGATCT				
			133R	ACCCATTCCTGCAACCTATGT				

Amplification efficiency and *r*^*2*^ depict average values. All qPCR assays were run using an annealing temperature of 64 °C.

*Housekeeping gene.

### Validation of housekeeping genes

Each qPCR run was accompanied by the quantitation of housekeeping genes for normalization. The genes *rnpA* (protein component of ribonuclease P) and *ppc* (phosphoenolpyruvate carboxylase) were selected as potential housekeeping genes (HKG) based on a previous qPCR study of *L. aestuarii*^26^. Validation of the stability of expression of these genes under different growth conditions was tested using the housekeeping gene determination software package BestKeeper^27^ of which standard deviation (SD) and correlation coefficient (*r*^2^) were used to verify HKG stability. According to the BestKeeper results for all treatments and light regimes, both HKGs revealed a stable expression in *L. aestuarii* (*rnpA*: SD =  ± 0.31–0.71, *r*^*2*^ = 0.89–0.99; *ppC*: SD =  ± 0.30–0.71, *r*^*2*^ = 0.90–0.99) and *C. chthonoplastes* (*rnpA*: SD =  ± 0.33–0.65, *r*^*2*^ = 0.83–0.96; *ppc*: SD =  ± 0.31–0.67, *r*^*2*^ = 0.87–0.95) and therefore both are suitable housekeeping genes for both species (Table 3).

**Table 3:** BestKeeper (HKG) *rnpA* and *pcc* of *L. aestuarii* and *C. chthonoplastes* in each treatment. Standard deviations (SD) and correlation coefficients (*r*^*2*^) are given.

HKG	Treatment	*L. aestuarii*		*C. chthonoplastes*	
		SD	*r*^*2*^	SD	*r*^*2*^
*rnpA*	ML	0.31	0.99	0.45	0.87
	CL	0.71	0.92	0.33	0.96
	MB	0.37	0.89	0.65	0.85
	CB	0.38	0.89	0.51	0.83
*ppc*	ML	0.3	0.99	0.43	0.87
	CL	0.71	0.9	0.32	0.95
	MB	0.37	0.92	0.67	0.89
	CB	0.4	0.96	0.53	0.87

### Effect of culturing on gene expression

**Figure 1:**
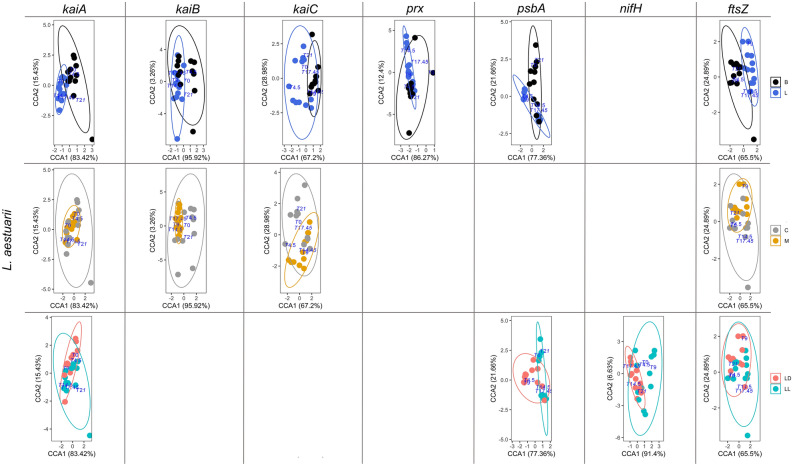
CCA plots of *kaiABC*, *prx*, *psbA*, *nifH* and *ftsZ* expression patterns in *L. aestuarii.* These genes were significantly influenced (*p* < 0.05, 95% confidence ellipse) by one or more of the treatments (L = liquid culture, B = biofilm-grown culture, M = mono culture, C = co-culture). Only conditions that were significantly affecting gene expression patterns are shown.

**Figure 2:**
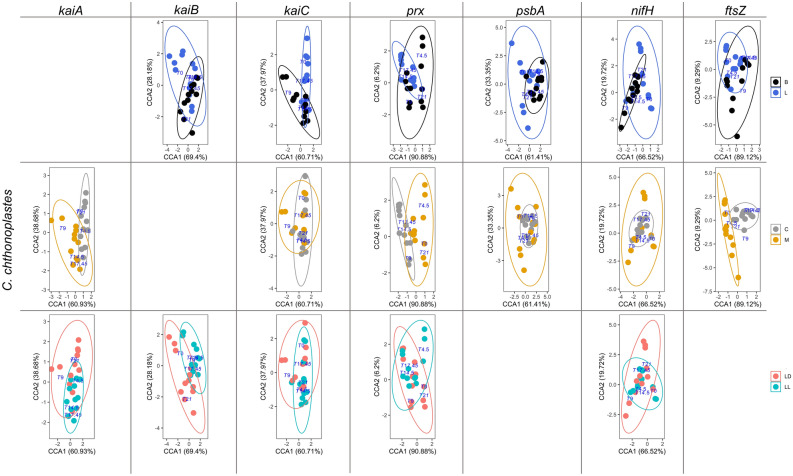
CCA plots of *kaiABC*, *prx*, *psbA*, *nifH* and *ftsZ* expression patterns of *C. chthonoplastes*. These genes were significantly influenced (*p* < 0.05, 95% confidence ellipse) by one or more of the treatments (L = liquid culture, B = biofilm-grown culture, M = mono culture, C = co-culture). Only conditions that were significantly affecting gene expression patterns are shown.

**Figure 3:**
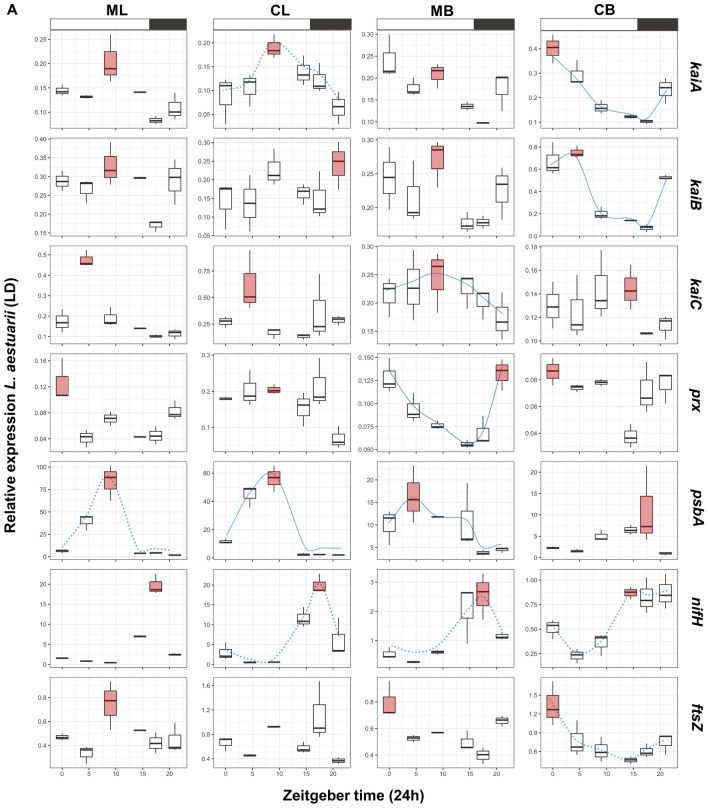

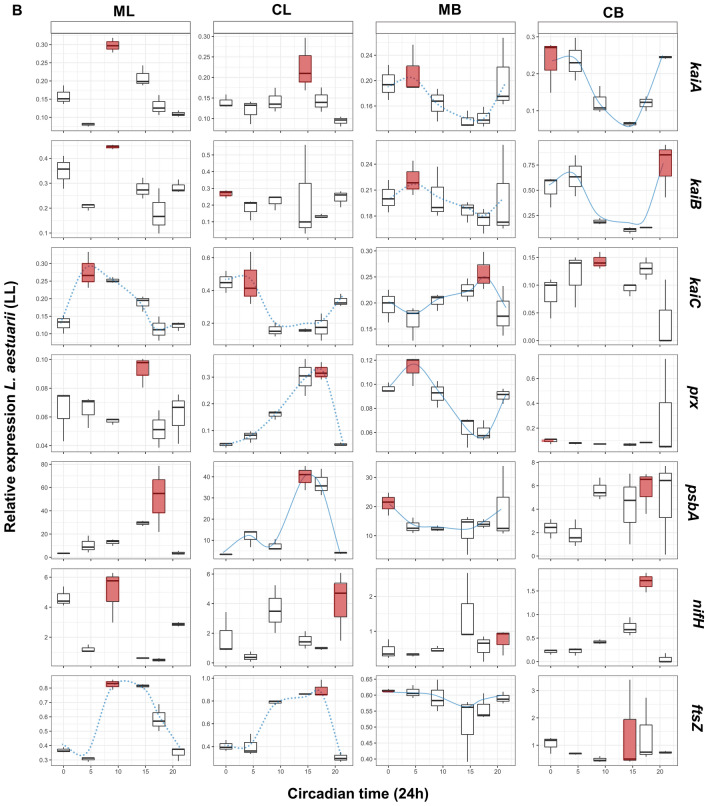
Boxplots presenting the relative normalized expression patterns in liquid mono- (ML) and co-cultures (CL) and biofilm-grown mono- (MB) and co-cultures (CB) of *L. aestuarii* under (**a**) LD and (**b**) LL regime. X-axes show Zeitgeber time (ZT: light–dark regime) (**a**) and circadian time (CT: constant light regime) (**b**). Red boxplots display the on average highest expression. Expressions patterns which display a blue (dotted) line display significant (*p* < 0.05) circadian rhythmicity as estimated by MetaCycle^29^. The dotted lines indicated significantly rhythmicity under LD illumination, while continuous lines symbolize significant rhythmicity under both light regimes (**a**, **b**). Boxplots display the minimum (lower error bar), maximum (upper error bar), median (horizontal line) and the first (box below the median) and third quartile (box above the median) of the expression values of the biological replicates (n = 3) per sample. A bar on top of the first row of the boxplots indicates the dark (black) and light (white) period (**a**). Treatments are distributed into columns and each row represents a gene.

**Figure 4:**
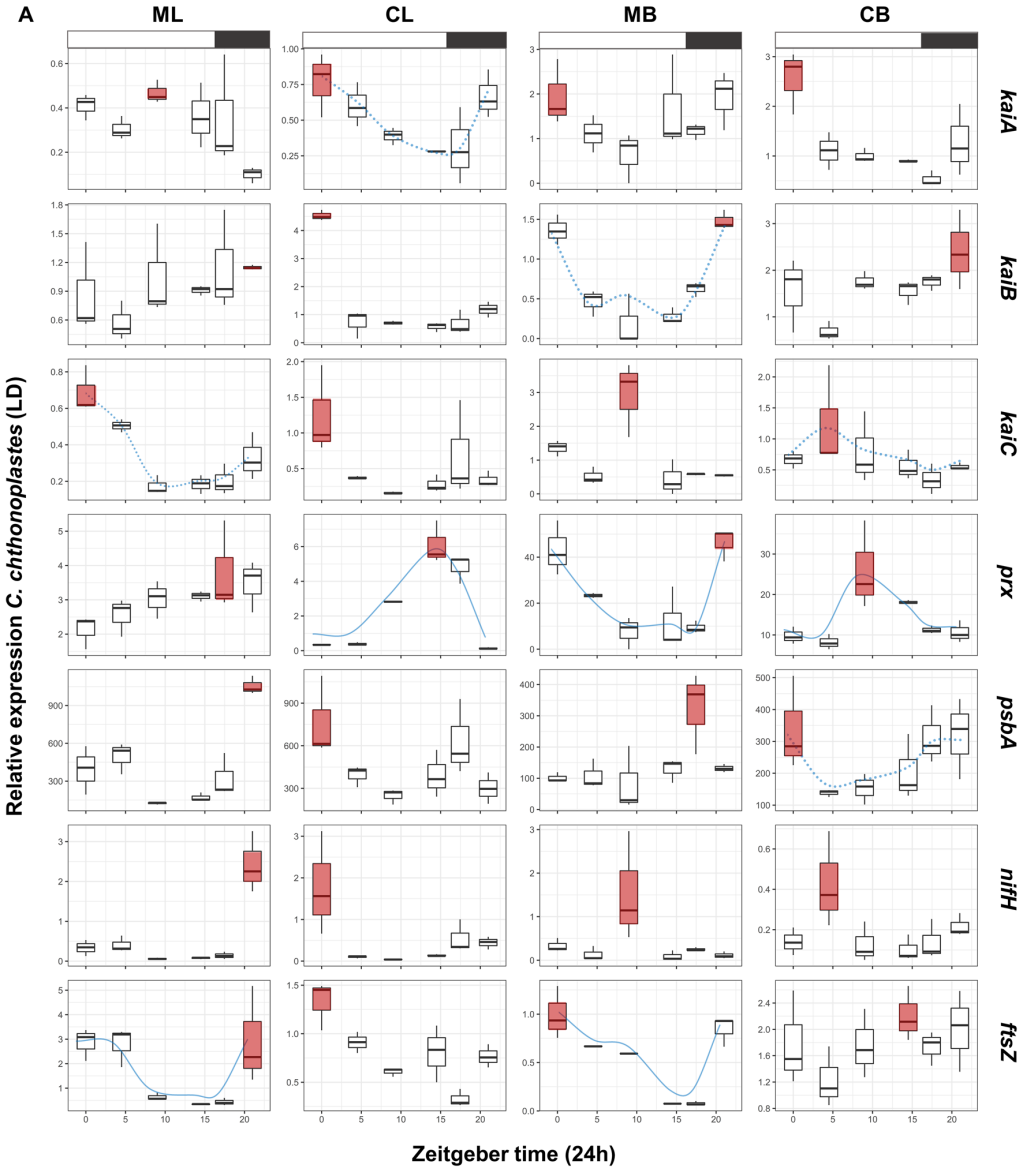

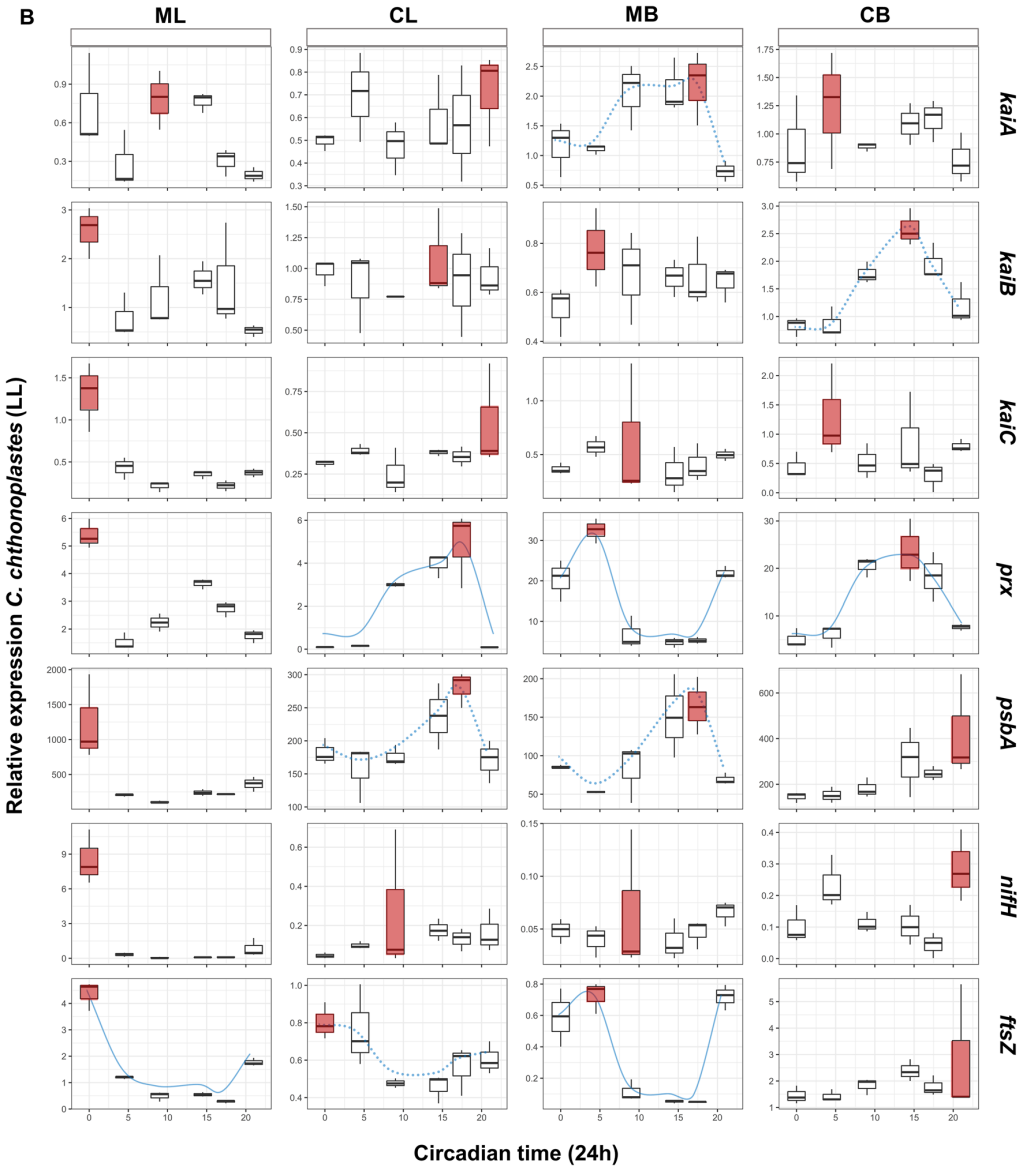
Boxplots presenting the relative normalized gene expression patterns in liquid mono-(ML) and co-cultures (CL) and biofilm-grown mono-(MB) and co-cultures (CB) of C. chthonoplastes under a (**a**) LD and (**b**) LL regime. X-axes show Zeitgeber (ZT: light–dark regime) (**a**) and circadian (CT: constant light regime) (**b**). Red boxplots display the on average highest expression. Expressions patterns which display a blue (dotted) line display significant (*p* < 0.05) circadian rhythmicity as estimated by MetaCycle^29^. The dotted lines indicated significantly rhythmicity under LD illumination, while continuous lines symbolize significant rhythmicity under both light regimes (**a**, **b**). Boxplots indicate the minimum (lower error bar), maximum (upper error bar), median (horizontal line) and the first (box below the median) and third quartile (box above the median) of the expression values of the biological replicates (n = 3) per sample. A bar on top of the first row of the boxplots indicates the dark (black) and light (white) period (**a**). A bar on top of the first row of the boxplots indicates the dark (black) and light (white) period (**a**). Treatments are distributed into columns and each row represents a gene.

A constrained correspondence analysis (CCA) was carried out in order to investigate the influence of culturing on the expression patterns of the target genes (Figs. 1 and 2 for *L. aestuarii* and *C. chthonoplastes,* respectively.). Housekeeping gene-normalized transcript levels at the different time points served as input data. The results of the CCAs were subjected to an analysis of variance like permutation test provided by the ‘vegan: Adonis’ package in R^28^ providing Chi-square (*Χ*^2^) and probability values (*p*(> F)) (*p* < 0.05) for each treatment (culture condition and light regime) (Table 4). For visualization purposes CCA plots were used to display data relationships (Figs. 1 and 2). For each strain, comparisons were performed relative to cells grown under “standard” laboratory conditions; i.e. liquid mono-cultures grown under LD light regime. Boxplots of the original data per gene per growth condition are presented in Figs. 3 and 4 for *L. aestuarii* and *C. chthonoplastes,* respectively. A direct comparison of the average gene expression levels between the different growth conditions is presented in supplementary figures S1A and B for *L. aestuarii* and *C. chthonoplastes,* respectively.

**Table 4:** Permutation (#999) test results for the constrained correspondence analysis. *Χ*^2^, F and *p* values are given for each cyanobacterial species, tested gene and way of cultivation. N = 24, degrees of freedom = 1.

Species	Gene	Treatments	ChiSquare	F	Pr(> F) (*p* < 0.05)
*L. aestuarii*	*kaiA*	mono_co	0.019847	3.3567	0.01
		LD_LL	0.017184	2.9064	0.02
		liquid_immobilized	0.074575	12.6132	0.001
	*kaiB*	mono_co	0.048847	12.8941	0.001
		LD_LL	0.001571	0.4147	0.744
		liquid_immobilized	0.033414	8.8203	0.002
	*kaiC*	mono_co	0.021277	4.6953	0.003
		LD_LL	0.008953	1.9757	0.083
		liquid_immobilized	0.03499	7.7215	0.001
	*psbA*	mono_co	0.012166	0.8989	0.401
		LD_LL	0.307779	22.7392	0.001
		liquid_immobilized	0.094425	6.9763	0.003
	*ftsZ*	mono_co	0.01304	4.07	0.012
		LD_LL	0.020866	6.5129	0.004
		liquid_immobilized	0.038839	12.1227	0.001
	*prx*	mono_co	0.19414	4.9258	0.057
		LD_LL	0.12722	3.2278	0.062
		liquid_immobilized	0.18735	4.7535	0.005
	*nifH*	mono_co	0.019527	2.2305	0.108
		LD_LL	0.296163	33.8281	0.001
		liquid_immobilized	0.012233	1.3972	0.191
*C. chthonoplastes*	*kaiA*	mono_co	0.077328	10.8547	0.001
		LD_LL	0.058531	8.2161	0.001
		liquid_immobilized	0.003109	0.4364	0.724
	*kaiB*	mono_co	0.002273	0.261	0.924
		LD_LL	0.0498	5.7195	0.002
		liquid_immobilized	0.040236	4.6211	0.009
	*kaiC*	mono_co	0.053274	4.4122	0.001
		LD_LL	0.039746	3.2918	0.007
		liquid_immobilized	0.083382	6.9058	0.001
	*psbA*	mono_co	0.0294	2.8843	0.029
		LD_LL	0.023192	2.2753	0.075
		liquid_immobilized	0.02871	2.8166	0.031
	*ftsZ*	mono_co	0.122411	18.9058	0.001
		LD_LL	0.006125	0.946	0.442
		liquid_immobilized	0.025181	3.8891	0.016
	*prx*	mono_co	0.263753	77.5317	0.001
		LD_LL	0.022111	6.4996	0.007
		liquid_immobilized	0.053278	15.6612	0.002
	*nifH*	mono_co	0.09931	3.0874	0.029
		LD_LL	0.21253	6.607	0.001
		liquid_immobilized	0.26686	8.296	0.001

### kaiABC expression in L. aestuarii

Comparison of the 24-h expression patterns of *kaiA*, *kaiB* and *kaiC* growing under different conditions (Fig. 1) revealed that their expression was significantly influenced by the way of culturing (liquid versus biofilm-grown growth; *Χ*^2^ = 0.033–0.074, *p* = 0.001–0.002) as well as whether co-cultured or as mono-culture (*Χ*^2^ = 0.019–0.212, *p* = 0.001–0.003) (Table 4) and explained ~ 81% of the observed variance in their expression. Unexpectedly, only *kaiA* was significantly influenced by the light regime (*Χ*^2^ = 0.017, *p* = 0.02) (Table 4). Potential rhythmic expression was deduced from comparing and fitting the expression patterns with a sine curve using the circadian cycle predictor program MetaCycle^29^ in accordance with a previous study in natural microbial mats^30^ (Supplementary Figure S2). Expression of *kaiA* followed a significant sinusoidal curve only in co-cultures under all growth conditions (*p* = 0.0084–0.01) and in biofilm-grown mono-culture under continuous illumination (*p* = 0.005–0.007) (Fig. 3a, b, Supplementary Figure S2). Highest expression of *kaiA* was observed in the light period, at point ZT9 in liquid mono- and co-cultures (ML & CL) and at ZT0 in biofilm-grown cultures (MB & CB) (Supplementary Figure S1A). A sinusoidal pattern for *kaiB* was only evident when biofilm-grown (LD-CB, LL-MB and LL-CB) (*p* = 0.006–0.02) (Fig. 3a, b, Supplementary Figure S2). Peak expression of *kaiB* varied with whether the organisms were co-cultured or not and ranged from ZT9 (ML, CL and MB) to ZT4.5 (CB). The highest peak expression measured for *kaiB* was observed in biofilm-grown co-cultures (Fig. 3a, Supplementary Figure S1A). Sinusoidal expression patterns for *kaiC* were mainly observed under continuous illumination (LL-ML, LL-CL and LL-MB) (*p* = 0.005–0.03) and in biofilm-grown mono-cultures under LD conditions (LD-MB) (*p* = 0.01) (Fig. 3a, b, Supplementary Figure S2). The time point of maximum *kaiC* expression (ZT4.5) was largely unaffected whether co-cultured or not but changed from ZT4.5 in liquid cultures to ZT9/ZT14.5 in biofilm-grown cultures. Under continuous illumination, a similar pattern was observed for *kaiC* and differences in peak expression were also only observed between liquid (peak at CT4.5) and biofilm-grown (peak at CT9) cultures (Fig. 3b, Supplementary Figure S1A).

### prx expression in L. aestuarii

Transcription of *prx* was overall low and was significantly affected when grown as liquid and biofilm-grown culture (*Χ*^2^ = 0.187, *p* = 0.005) (Table 4). A sinusoidal pattern was observed for biofilm-grown mono-cultures (MB) under LD illumination (*p* = 0.01) and for CL and MB under continuous illumination (*p* = 0.004–0.01) (Fig. 3a, b, Supplementary Figure S2). Highest expression of *prx* was observed in the late dark/early light period (ZT21, ZT0) in liquid mono-cultures and in biofilm-grown mono- and co-cultures. These expression peaks shifted in liquid cultures, grown under continuous light, to CT14.5 (ML) and CT17.45 (CL) and to CT4.5 (MB) and CT0 (CB) in biofilm-grown cultures (Supplementary Figure S1A).

### psbA expression in L. aestuarii

The variation of expression of *psbA* in *L. aestuarii* during a 24-h period differs significantly between different light regimes (LD versus-LL; *Χ*^2^ = 0.307, *p* = 0.001) and the way of cultivation (biofilm-grown versus liquid; *Χ*^2^ = 0.094, *p* = 0.003) (Fig. 1, Table 4). Cultures growing under a light–dark regime revealed a sinusoidal pattern of *psbA* gene expression (*p* = 0.007–0.02) (Fig. 3a, b, Supplementary Figure S2) with the exception of CB cultures. Highest *psbA* expression was found during the beginning of the light period (ZT4.5-ZT9) followed by a sharp drop in expression in liquid cultures at ZT14.5 (Fig. 3a, b, Supplementary Figure S1A). In biofilm-grown cultures the overall expression level of *psbA* was lower than in the liquid cultures, while in CB cultures the highest expression was at the end of the light, beginning of the dark period (ZT14.5-ZT17.45). Under continuous illumination the overall expression levels in liquid cultures were slightly lower and a shift in expression pattern was observed with highest expression at ZT14.5-ZT17.45 (Fig. 3b, Supplementary Figure S2). The sinusoidal pattern was maintained under continuous illumination for CL and MB conditions biofilm-grown (*p* = 0.01–0.04) (Fig. 3a, b, Supplementary Figure S2).

### nifH expression in L. aestuarii

Under the standard dark–light regime, *nifH* expression was low during the light and was highest during the dark (ZT17.45-ZT21) (Fig. 3a, Supplementary Figure S1A). Moreover, *nifH* expression was significantly higher in liquid cultures compared to the biofilm-grown cultures. A significant sinusoidal curve under LD illumination was observed under all conditions (*p* = 0.005–0.01) except for the co-cultured biofilm-grown cultures (CB) (Fig. 3a, Supplementary Figure S2). The imposed light regime significantly affected the daily transcription pattern of *nifH* (*Χ*^2^ = 0.296, *p* = 0.001) (Fig. 1, Table 4). Under continuous light, the pattern of expression in the liquid cultures peaked more often than in biofilm-grown cultures with high expression at CT0, CT4.5 and CT21. (Fig. 3b, Supplementary Figure S1A). In biofilm-grown mono-cultures *nifH* expression peaked at CT14.5 and CT21 and in co-cultures at CT17.45. No significant sinusoidal nifH gene expression was found under continuous illumination.

### ftsZ expression in L. aestuarii

The variation in expression of *ftsZ* in *L. aestuarii* during a 24-h period was large and only in co-cultured biofilm-grown cultures at LD illumination a sinusoidal like curve was observed with a maximum at ZT0 (*p* = 0.03) (Fig. 3a, Supplementary Figure S2). In contrast, under continuous illumination an opposite pattern is found with all the three other growth conditions providing significant sinusoidal *ftsZ* expression (*p* = 0.008–0.01) (Fig. 3b, Supplementary Figure S1A). Due to other more variable patterns, each of the imposed cultivation conditions appeared to contribute significantly to the gene expression pattern (liquid vs. biofilm-grown: *Χ*^2^ = 0.038, *p* = 0.001; LD/LL: *Χ*^2^ = 0.020, *p* = 0.004 and mono-/co-culture: *Χ*^2^ = 0.013, *p* = 0.012) (Fig. 1, Table 4).

### kaiABC expression in C. chthonoplastes

The expression pattern of *kaiA*, *kaiB* and *kaiC* of *C. chthonoplastes* grown under a LD regime changed significantly when transferred to continuous light (*Χ*^2^ = 0.049–0.058, *p* = 0.001–0.007) (Fig. 2, Table 4). Furthermore, the expression of *kaiA* and *kaiC* differed when the organism was grown as a mono-culture or co-cultured with *L. aestuarii* (mono- vs. co-culture: *Χ*^2^ = 0.053–0.077, *p* = 0.001), while the way of culturing significantly impacted on the expression of *kaiB* and *kaiC* during a 24-h period (liquid- vs. biofilm-grown-culture: Χ^2^ = 0.040–0.833, p = 0.001–0.009) (Fig. 2, Table 4).

The expression of *kaiA* and *kaiB* did not seem to follow a particular pattern and only in liquid co-cultures a potential sinusoidal curve was discerned for *kaiA* expression (*p* = 0.005) and in biofilm-grown mono-cultures for *kaiB* (*p* = 0.03) (Fig. 4a, Supplementary Figure S1). Peak expression for *kaiA* in liquid co-cultures and biofilm-grown cultures was at the beginning of the light period (ZT0) and at the end of the dark period (ZT21) and peaked at ZT0 and ZT9 in liquid mono-cultures (Fig. 4a). A sinusoidal distribution was also observed for *kaiC* expression of cells grown in ML and CB conditions with respective highest expression at ZT0 and ZT4.5 (*p* = 0.01) (Fig. 4a, Supplementary Figure S2).

Under continuous illumination, *kaiA* expression was sinusoidal at LL- MB (*p* = 0.01) and *kaiB* at LL-CB (*p* = 0.003) with peak expression at CT17.5 and CT14.5 (Fig. 4b, Supplementary Figure S2). Potential rhythmic expression of *kaiC* under LL conditions was not observed.

### prx expression in C. chthonoplastes

The expression pattern of *prx* in *C. chthonoplastes* differed significantly between a mono-culture and co-culture with *L. aestuarii* (*Χ*^2^ = 0.263, *p* = 0.001), and between a liquid medium and biofilm-grown culture (*Χ*^2^ = 0.053, *p* = 0.002), as well as upon transfer to continuous light (*Χ*^2^ = 0.022, *p* = 0.007) (Fig. 2, Table 4). Overall, the expression of *prx* was 5- (LL) to tenfold (LD) higher in biofilm-grown than in liquid culture (Fig. 4a, b, Supplementary Figure S1B). Expression of *prx* under standard conditions (ML-LD) gradually increased from the beginning of light to the dark period while an almost opposite trend was observed in MB cultures with highest expression at time points ZT0 and ZT21. A sinusoidal pattern was observed in the co-cultures and in biofilm-grown mono-cultures independent of the light regimes tested (*p* = 0.006–0.04) (Fig. 4a, b, Supplementary Figure S2).

### psbA expression in C. chthonoplastes

Expression of *psbA* in *C. chthonoplastes* was significantly influenced by two of the growth conditions (mono-/co-culture: *Χ*^2^ = 0.029, *p* = 0.029, liquid vs. biofilm-grown: *Χ*^2^ = 0.028, *p* = 0.031) (Fig. 2, Table 4). A sinusoidal pattern was observed for *psbA* under CB conditions at LD illumination (*p* = 0.01) and under CL and MB conditions under continuous illumination (*p* = 0.003–0.01) (Fig. 4a, b, Supplementary Figure S2). The expression of *psbA* in LD-grown cultures peaked during the early dark to early light period (ZT17.45-ZT0) (Fig. 4a, Supplementary Figure S1B). Under continuous illumination, co-cultures and MB cultures revealed peaks in the expression of *psbA* between CT14.5 and CT21, while the ML culture was characterized by high *psbA* expression at CT0 (Fig. 4b, Supplementary Figure S1B).

### nifH expression in C. chthonoplastes

The expression of *nifH* was significantly affected by the way of culturing (liquid vs. biofilm-grown: *Χ*^2^ = 0.266, *p* = 0.001, mono-/co-culture: *Χ*^2^ = 0.099, *p* = 0.029) and by transfer to constant light (*Χ*^2^ = 0.212, *p* = 0.001) (Fig. 2, Table 4). Relative *nifH* expression in biofilm-grown, co-culture-grown and LL-grown cultures displayed less fluctuation than displayed in liquid-grown, mono-culture-grown and LD-grown cultures. Expression of *nifH* in *C. chthonoplastes* was overall low with a single peak at the late dark/early light period in liquid cultures (ZT21 (ML), ZT0 (CL)) and during the light period in biofilm-grown cultures (ZT9 (MB), ZT4.5 (CB)) (Fig. 4a, Supplementary Figure S1B). In the liquid mono-culture at LL a single peak in the expression of *nifH* was observed at CT0 (Fig. 4b, Supplementary Figure S1B). No significant sinusoidal patterns were found for *C. chthonoplastes nifH* expression under any of the tested conditions.

### ftsZ expression in C. chthonoplastes

Expression of the cell division protein coding gene *ftsZ* was significantly affected whether co-cultured or not (*Χ*^2^ = 0.122, *p* = 0.001) and liquid- or biofilm-grown culture (*Χ*^2^ = 0.025, *p* = 0.016) (Fig. 2, Table 4). The patterns of expression of *ftsZ* during a 24-h period varied. A sinusoidal curve was only observed in mono-cultures (*p* = 0.006–0.01) and at LL-CL and LL-MB (*p* = 0.008–0.04) (Fig. 4a, b, Supplementary Figure S2) with peak expression during the late dark and early light period in LD-grown cultures and at same time points in LL-grown cultures. (Fig. 4a, b, Supplementary Figure S1B).

## Discussion

### The circadian clock of *L. aestuarii* and *C. chthonoplastes*

Rhythmic gene expression in cyanobacteria involves the following three steps: (1) the external cues, mostly light, temperature and redox potential that (2) entrain the “clock” proteins that control the rhythmic expression of (3) the functional protein coding genes involved in processes that are separated in time.

The only controlled rhythmic cue in our experimental set-up was the illumination period while temperature was kept the same in all experiments. Given the large number of variables tested within one cycle and the need for at least 6 time points per cycle, we were not able to assay over a 48 h or longer period that would be needed to determine truly circadian control. However, the observed significant differences in expression patterns are sufficient to infer that circadian control may also be affected by the greater complexity of natural conditions.

Both the way of culturing (liquid or biofilm-grown) as well as whether the cyanobacteria were co- or mono-cultured affected the expression of the circadian clock genes *kaiA*, *kaiB*, and *kaiC* in both species, albeit in different ways.

The maximum expression of the circadian clock genes *kaiABC* of *L. aestuarii* shifted 4–8 h between liquid and biofilm-grown cultures. Shifting the cultures to continuous illumination for 2 days, from which samples were taken on the second day, had little influence on the variation of expression of the three *kai* genes over a 24-h period, which is indicative of the free-running cyanobacterial clock as has been previously shown for unicellular cyanobacteria^31,32^. In contrast, the cultures of *C. chthonoplastes* did not display a free-running clock. Under continuous light, maximum gene expression of *kaiABC* in this organism shifted in regard to what has been found under a dark–light regime when grown in biofilm-grown cultures and co-culture.

Based on the aforementioned results we suggest that, despite being both members of the order of Oscillatoriales, the two species display major differences in how stringent the control over their circadian clocks is. These differences may be attributed to the life strategies of the two cyanobacteria regarding their response to high light. For example, *C. chthonoplastes* avoids light stress by migration^33,34^, which may trigger several transcriptional responses in line with a metabolism that is not optimally adapted to continuous high light intensities. In contrast, *Lyngbya* only moves when differentiating into hormogonia: short, sheet-less motile trichomes^35^, and therefore cannot rapidly avoid the imposed light stress. However, *Lyngbya* species evolved strategies to endure high light, desiccation and UV-stress. To avoid the latter, *Lyngbya* species synthesize UV-screening compounds such as mycosporine amino acid-like substances (MAAs) and carotenoids, in addition to coiling of their trichomes^36^. These strategies may not require additional transcriptional regulation in response to continuous illumination and might explain why the expression of *kaiABC* in *Lyngbya* sp. does not differ between the two illumination set ups. The observed expression patterns in *C. chthonoplastes* are less well understood and perhaps appear more similar to an ‘hourglass’ clock, which has been described for *kaiA*-lacking species such as *Prochlorococcus marinus*^37^. However, since *C. chthonoplastes* does have, and expresses the *kaiA* gene, the differences in expression with *L. aestuarii* may reflect specific adaptation to their natural habitat. In support of this assumption is the low identity between *kaiA* nucleotide and protein sequences (< 60% identity) of *L. aestuarii* and *C. chthonoplastes* (and also compared to the *kaiA* sequence of *S. elongatus*), which is also the lowest in comparison to *kaiB* and *kaiC* (> 75% identity), suggesting that *kaiA* is the least conserved gene and may experience the lowest selective pressure among the *kai* genes (Supplementary Table S1, Supplementary Figure S3). Even though the two species co-occur in many marine microbial mats, *L. aestuarii* and *C. chthonoplastes* may occupy different ecological niches that allow them to share light and micronutrients using differently tuned circadian clocks.

A *kaiABC*-independent circadian oscillator has been linked to the periodicity of peroxiredoxin which follows the generation of stress-induced reactive oxygen species^25,38^. Overall, the expression patterns of *prx* reveal a significant sinusoidal curve over the 24-h sampling period for most conditions except the standard liquid mono-cultures in *C. chthonoplastes* and under some conditions in *L. aestuarii*. However, the expression levels of *prx* shifted significantly between mono- and co-cultures of liquid (*L. aestuarii*) and biofilm-grown (*C. chthonoplastes*) cultures. Highest *prx* expression was found in mono-cultures in the dark to early light period. This was also found in *Crocosphaera watsonii* strain WH8501 and it was attributed to the accumulation of reactive oxygen species (ROS) towards the end of the light period^39^. In contrast to mono-cultures, *prx* expression in co-cultures of *L. aestuarii* and *C. chthonoplastes* displayed maxima towards the late light period. This would suggest that the accumulation of ROS occurs faster in co-cultures as the result of a higher raise of oxygen concentration due to the combined photosynthetic activity. Robust free-running *prx* expression^25^ without a peak shift was only found in co-cultures of liquid (*C. chthonoplastes*) and biofilm-grown (*L. aestuarii*) cultures under continuous illumination. Regardless of the illumination regime and treatment, average *prx* levels of *C. chthonoplastes* were more than a 100-fold higher than in cultures of *L. aestuarii* and showed a tenfold increase in biofilm-grown cultures in comparison to liquid cultures. The large transcriptional discrepancy between the species’ *prx* levels and between *C. chthonoplastes*’ liquid and biofilm-grown cultures suggests a higher sensitivity of *C. chthonoplastes* and especially of its biofilm-grown cultures, to oxidative stress and explains its migratory behaviour to avoid high light intensities.

### The expression of circadian clock regulated genes in mono- and co-cultures grown in free-living or biofilm-grown mode

Depending on growth conditions, expression of *psbA* in *C. chthonoplastes* and *L. aestuarii*, and *nifH* in *L. aestuarii* followed a sinusoidal pattern during the 24-h period. In the unicellular cyanobacteria *Cyanothece* sp., expression of *psbA* and *nifH* is under control of the *kaiABC* regulatory network^40^ similar as for *psbA* expression in *S. elongatus*^41^. The peak in expression of *psbA* in the early light period and of *nifH* in the dark is typical for non-heterocystous, circadian clock controlled cyanobacteria in which nitrogenase activity is confined to the low light periods where photosynthetically produced oxygen is low^42,43^. Remarkably, we did not find such distinction between the peaks of *psbA* and *nifH* expression in *L. aestuarii* grown as a biofilm-grown co-cultured with *C. chthonoplastes*. Instead, both *psbA* and *nifH* peaked during the dark. It is unclear why the expression of *psbA* shifted to the dark in biofilm-grown co-cultures. Moreover, under LD conditions *psbA* and *nifH* peak transcription levels in *L. aestuarii* were up to 11 times lower in biofilm-grown cultures compared to liquid cultures. Albeit less distinct, the same was seen in *C. chthonoplastes*. The lower expression of *psbA* in biofilm-grown cultures may have been caused by the lower exposure to light of the biofilm-grown trichomes or by a decreased rate of growth as has been observed in aggregated cells in biofilms of *Escherichia coli*^44^. However, the latter explanation was not supported by lower transcription levels of the cell division gene *ftsZ* in biofilm-grown cultures. Alternatively, as is the case in *S. elongatus*, reactive oxygen species may target the de novo synthesis of *psbA* mRNA^45,46^ decreasing the rate of photosynthesis^47^. This explanation is supported by the increase of *prx* expression in biofilm-grown cultures that is possibly a response to an increase in ROS.

When *L. aestuarii* was transferred to continuous illumination the expression peak of *psbA* shifted 4–8 h later during the 24-h cycle. In contrast, *C. chthonoplastes*, which does not display a free-running circadian rhythm, the peak of the expression of *psbA* did not shift when the culture was transferred to constant illumination. These results challenge the degree of control of the circadian clock on the expression of *psbA* in these cyanobacteria and call for another controlling mechanism.

The measurement of expression of *nifH* in *C. chthonoplastes* was hindered by technical difficulties in the qPCR reaction yielding in some runs false positive reactions above the threshold level in the non-template controls, which was attributed to a known reagent contamination problem^48^. However, this problem is mostly neglectable when large amounts of species specific *nifH* containing DNA is added as template. With some caution we conclude that *nifH* expression in *C. chthonoplastes* revealed low transcript numbers and lack of temporal separation from *psbA* expression. This is unusual for non-heterocystous cyanobacteria. Moreover, it is still uncertain whether *C. chthonoplastes* actually fixes atmospheric dinitrogen since it has not yet been possible to grow the species diazotrophically in culture^49^. Although this cyanobacterium possesses the *nifHDK gene cluster*^50^, it lacks the accessory genes *nifOTWXZ*, which are present in, for instance, *L. aestuarii*^51^ . The function of these accessory *nif* genes is not well understood and it is therefore not certain whether they are indispensable for N_2_ fixation. Daily patterns of *nifH* expression in biofilm-grown *C. chthonoplastes* cultures are similar to those observed in a coastal microbial mat^52^ as well as metatranscriptomes from the same mat^30^. These studies report that *nifH* expression is high in the beginning and/ or middle of the light period.

In conclusion, comparison of gene expression patterns between laboratory cultures and field samples are rare and may be contradictory. The daily variations of gene expression obtained from the metatranscriptome of oceanic surface water containing abundant picocyanobacteria (*Synechococcus*) were similar to those obtained from liquid cultures^53^. The laboratory conditions for liquid cultures may better represent the natural conditions of these planktonic species than would be the case for benthic microorganisms. The transcriptomes of *Salinibacter ruber* and *Haloquadratum walsbyi* reveal large differences in relative expression levels in a subset of genes while relative expression levels of other genes are well conserved in the metatranscriptomes from natural samples in which these organisms thrive (Bolhuis, unpublished data).

Our study shows that gene expression in two filamentous non-heterocystous cyanobacteria respond differently to external stimuli and potential zeitgebers. Phase shifts as well as changes in expression levels occurred as a function of the mode of growth (liquid medium versus biofilm-grown culture) and to the presence of the other competitor species. The observed species-specific expression patterns most likely reflect different life strategies, hinted at by differences in expression of *prx*, *psbA*, and *nifH*. In this study, neither co- or mono-cultivation nor liquid or biofilm-grown growth could be held as main responsible effector for the observed differences in gene expression patterns. However, in combination these effectors are able to alter gene expression not only in the laboratory but also in nature. As a consequence, most laboratory derived cyanobacterial circadian clock (controlled) expression patterns are insufficient predictors for expression patterns in the field. This emphasizes the need to mimic the natural environment when testing bacteria in the laboratory.

## Material and methods

### Experimental setup

Stock cultures of the filamentous, non-heterocystous cyanobacteria *Lyngbya aestuarii* PCC8106 (synonym = *Lyngbya* sp*.* CCY9616) and *Coleofasciculus* (*Microcoleus*) *chthonoplastes* PCC7420 (CCY9604) served as inoculum for the experiments. These cyanobacteria were isolated from similar coastal microbial mats but at different times and at a large geographical distance (Mellum, Germany (*L. aestuarii*) and Woods Hole, USA (*C. chthonoplastes*)). Although the two species, both members of the order Oscillatoriales, are naturally co-occurring in coastal microbial mats and in many other similar habitats^54–57^, the isolates tested here did not have any previous interaction other than the three weeks of co-culturing for the experiments described here. The cyanobacteria were grown at 23 °C under continuous fluorescent light (L: photon density: 40 µmol m^−2^ s^−1^) in 40 ml culture flasks (TPP, Switzerland) containing 25 ml of BA + medium (1:1 mix of BG11 and ASN 3) (www.dsmz.de) with added nitrate ( +) (13.29 mM). The culturing was done in triplicate. Liquid cultures (L) were grown in the 40 ml TP culture flasks. Biofilm-grown cultures (B) were grown in 6-well culture plates equipped with filter trays (0.4 µm pore size) (NUNC™, Denmark), which were filled with sterile glass beads (3.5 g/well) (ø 0.1 mm) and 4 ml/well of BA + medium (which did not submerse the beads). Sterile tweezers and disposable sterile Pasteur pipettes (VWR, USA) were used to distribute equally sized pieces of cyanobacterial trichomes on the glass beads in order to obtain an even distribution. Co-culturing (C) was done by inoculating similar sized filaments of both species (1:1 ratio) in culture flasks or by placing their trichomes on the glass bead surface. The cultures were grown for three weeks under a 16 h light and 8 h dark cycle (LD) to entrain the circadian clock. Two days prior to sampling, half of the LD cultures of each treatment was exposed to constant light (LL) in order to test the free-running characteristics of the circadian clock. During the three-week culturing period, the cyanobacteria did not show signs of degradation or bleaching. Evaporation of medium during culturing was compensated by refilling wells and culture flasks with autoclaved MilliQ water.

### Sampling and RNA extraction

RNA was extracted from *L. aestuarii* and *C. chthonoplastes* cultures grown in liquid medium (L) or biofilm-grown on glass beads (B) as mono- (M) or as co-culture (C). Sampling of cell material occurred at 6 time points during a light–dark regime (LD) indicated with the Zeitgeber Time (ZT) ZT0 (05:00 h), ZT4.5 (09:30 h), ZT9 (14:00 h), ZT14.5 (19:30 h), ZT17.45 (22:45 h) and ZT21 (02:00 h). Cultures transferred to continuous light (LL) were sampled at the corresponding time points of the LD series but were labelled as Circadian Time (CT). Samples were taken in biological triplicates and immediately submerged in liquid nitrogen in a sterile mortar and ground with a sterile pestle. From the liquid cultures an equal number of subsamples were taken and from the biofilm-grown samples a complete well with cell material was used for RNA extraction. After grinding, the material was placed in bead tubes provided by the ZR-Fungal/ Bacterial RNA MiniPrep isolation kit (ZYMO research, USA) and quickly frozen in liquid nitrogen prior to short-term storage at − 80 °C. RNA extraction was done according to the manufacturers’ protocol. Quantity, quality, and efficiency of DNA removal was measured after DNAse treatment of each RNA sample (TurboDNAse, LifeTechnology, USA) by using a Bioanalyzer 2,100 (Agilent Technologies, USA). Three µl of each of the DNAse treated samples containing 2 ng/µl RNA (RIN > 5) were reverse transcribed into cDNA using SuperScript III reverse transcriptase (200U/µl) and random hexamer primers (100 ng/μl) followed by an RNase treatment to remove residual RNA according to the manufacturers’ protocol (Life Technologies, USA).

### Gene quantification

Based on the genome sequences of *L. aestuarii* and *C. chthonoplastes*, primers and TaqMan probes of the targeted circadian clock gene cluster *kaiABC*, the circadian clock reporter gene *cikA*, nitrogenase gene *nifH*, cell division protein gene *ftsZ*, photosynthesis D1 protein gene *psbA*, peroxiredoxin gene *prx* and two commonly used cyanobacterial housekeeping genes, *rnpA* and *ppc*^26^, were designed using the genetic analysis software Geneious R 8.1.7^58^ (Table 2). The proper annealing temperatures of the primers were established by gradient PCR on a thermocycler (BioMetra) and specificity was confirmed by Sanger sequencing of the amplicons (BaseClear, Leiden, The Netherlands). Standard curves for RT-qPCR were prepared by using dilution series of PCR products and the primers were checked for species specificity by means of crossover PCRs and gel electrophoreses. Samples, standard curves and non-template controls were run in technical triplicates on a Rotor-Gene 6000™ (Qiagen, USA).

RT-qPCR amplification of the targeted and housekeeping genes was performed in 15 µl volumes containing 2.25 µl MilliQ, 7.5 µl 2 × Multiplex qPCR Perfecta Supermix (Quanta Biosciences), 0.38 µl (0.5 µM) of each target and housekeeping gene primer, 0.19 µl of each probe (0.25 µM) and 1.5 µl of template. RT-qPCR cycling was performed at an initial activation step of 95 °C for 2 min followed by 40 cycles at 95 °C for 10 s and at 64 °C for 60 s. Gene amplification efficiencies (%), *r*^2^, Ct values and transcript abundances (copies/µl) were retrieved from the program Rotor-Gen Q 2.1.0 (Qiagen) (Supplementary Table S2). Where needed, missing Ct values of biological replicates were surrogated by the median of the remaining biological replicates^59^.

### Statistics

To evaluate the validity of the housekeeping genes across treatments, Ct values of genes of interest and housekeeping genes were used in the program BestKeeper^27^. T-tests were applied to examine the sample-wise expression within each genes’ expression profile and the influence of treatments on transcription was assessed using R-based CCA scripts and ANOVA (Χ^2^, F- and *p *values (< 0.05)). In these tests normalized transcript abundances (($\frac{GOI }{geomean(HKGs)}$)) of each gene served as input data. CCAs were plotted and supplemented with confidence ellipses (95%) of the standard expression of the different genes. Potential significant rhythmic gene expression following a sinusoidal curve was predicted by the R based script Metacycle^29^ using the transcription levels at different time points as input matrix. The cycle analysis was performed with the meta2D function that integrates multiple cycle prediction methods and combines their *p*-values. Training of the cycling algorhythm was performed by combining the datasets, thereby mimicking a 72 h sampling pattern and a p-value cut-off of 0.05 was used to determine potential sinusoidal patterns.

## Supplementary information

Supplementary information

## References

[1] De Roy K, Marzorati M, Van den Abbeele P, Van de Wiele T, Boon N (2014). Synthetic microbial ecosystems: an exciting tool to understand and apply microbial communities. Environ. Microbiol..

[2] Mee MT, Wang HH (2012). Engineering ecosystems and synthetic ecologies. Mol. Biosyst..

[3] Hendrickx L (2006). Microbial ecology of the closed artificial ecosystem MELiSSA (micro-ecological life support system alternative): reinventing and compartmentalizing the Earth’s food and oxygen regeneration system for long-haul space exploration missions. Res. Microbiol..

[4] Chen Y (2011). Development and application of co-culture for ethanol production by co-fermentation of glucose and xylose: a systematic review. J. Ind. Microbiol. Biotechnol..

[5] Spus M (2015). Strain diversity and phage resistance in complex dairy starter cultures. J. Dairy Sci..

[6] Ma Q (2011). Integrated proteomic and metabolomic analysis of an artificial microbial community for two-step production of vitamin C. PLoS ONE.

[7] Petrof EO (2013). Stool substitute transplant therapy for the eradication of *Clostridium difficile* infection: ‘RePOOPulating’ the gut. Microbiome.

[8] Bolhuis H, Stal LJ (2011). Analysis of bacterial and archaeal diversity in coastal microbial mats using massive parallel 16S rRNA gene tag sequencing. ISME J..

[9] van Gemerden H (1993). Microbial mats: a joint venture. Mar. Geol..

[10] Tolker-Nielsen T, Molin S (2000). Spatial organization of microbial biofilm communities. Microb. Ecol..

[11] Bolhuis H, Cretoiu MS, Stal LJ (2014). Molecular ecology of microbial mats. FEMS Microbiol. Ecol..

[12] Cohen SE, Golden SS (2015). Circadian rhythms in cyanobacteria. Microbiol. Mol. Biol. Rev..

[13] Nakajima M (2005). Reconstitution of circadian oscillation of cyanobacterial KaiC phosphorylation in vitro. Science.

[14] Murayama Y (2017). Low temperature nullifies the circadian clock in cyanobacteria through Hopf bifurcation. Proc. Natl. Acad. Sci. USA..

[15] Johnson CH, Zhao C, Xu Y, Mori T (2017). Timing the day: what makes bacterial clocks tick?. Nat. Rev. Microbiol..

[16] Woelfle MA, Ouyang Y, Phanvijhitsiri K, Johnson CH (2004). The adaptive value of circadian clocks: an experimental assessment in cyanobacteria. Curr. Biol..

[17] Welkie DG (2019). A hard day’s night: cyanobacteria in diel cycles. Trends Microbiol..

[18] Ivleva NB, Gao T, LiWang AC, Golden SS (2006). Quinone sensing by the circadian input kinase of the cyanobacterial circadian clock. Proc. Natl. Acad. Sci. USA..

[19] Gutu A, O’Shea EK (2013). Two antagonistic clock-regulated histidine kinases time the activation of circadian gene expression. Mol. Cell.

[20] Rust MJ, Golden SS, O’Shea EK (2011). Light-driven changes in energy metabolism directly entrain the cyanobacterial circadian oscillator. Science.

[21] Pattanayak G, Rust MJ (2014). The cyanobacterial clock and metabolism. Curr. Opin. Microbiol..

[22] Mackey SR, Golden SS, Ditty JL (2011). The itty-bitty time machine genetics of the cyanobacterial circadian clock. Adv. Genet..

[23] Resch A, Rosenstein R, Nerz C, Götz F (2005). Differential gene expression profiling of *Staphylococcus aureus* cultivated under biofilm and planktonic conditions. Appl. Environ. Microbiol..

[24] Moorthy S, Watnick PI (2005). Identification of novel stage-specific genetic requirements through whole genome transcription profiling of *Vibrio cholerae* biofilm development. Mol. Microbiol..

[25] Edgar RS (2012). Peroxiredoxins are conserved markers of circadian rhythms. Nature.

[26] Pinto F, Pacheco CC, Ferreira D, Moradas-Ferreira P, Tamagnini P (2012). Selection of suitable reference genes for RT-qPCR analyses in cyanobacteria. PLoS ONE.

[27] Pfaffl MW, Tichopad A, Prgomet C, Neuvians TP (2004). Determination of stable housekeeping genes, differentially regulated target genes and sample integrity: Bestkeeper–Excel-based tool using pair-wise correlations. Biotechnol. Lett..

[28] Oksanen, J. *et al. Package ‘vegan’ Title Community Ecology Package*. *Community Ecol. Packag.* 2**,** (2019).

[29] Wu G, Anafi RC, Hughes ME, Kornacker K, Hogenesch JB (2016). MetaCycle: an integrated R package to evaluate periodicity in large scale data. Bioinformatics.

[30] Hörnlein C, Confurius-Guns V, Stal LJ, Bolhuis H (2018). Daily rhythmicity in coastal microbial mats. npj Biofilms Microbiomes.

[31] Kondo T (1993). Circadian rhythms in prokaryotes: luciferase as a reporter of circadian gene expression in cyanobacteria. Proc. Natl. Acad. Sci. USA..

[32] Tomita J (2005). No transcription-translation feedback in circadian rhythm of KaiC phosphorylation. Science.

[33] Whale GF, Walsby AE (1984). Motility of the cyanobacterium *Microcoleus chthonoplastes* in mud. Br. Phycol. J..

[34] Urmeneta J, Navarrete A, Huete J, Guerrero R (2003). Isolation and characterization of cyanobacteria from microbial mats of the Ebro Delta Spain. Curr. Microbiol..

[35] Kothari A, Vaughn M, Garcia-Pichel F (2013). Comparative genomic analyses of the cyanobacterium, *Lyngbya aestuarii* BL J, a powerful hydrogen producer. Front. Microbiol..

[36] Rath J, Adhikary SP (2007). Response of the estuarine cyanobacterium *Lyngbya aestuarii* to UV-B radiation. J. Appl. Phycol..

[37] Holtzendorff J (2008). Genome streamlining results in loss of robustness of the circadian clock in the marine cyanobacterium *Prochlorococcus marinus* PCC 9511. J. Biol. Rhythms.

[38] O’Neill J, Reddy A (2011). Circadian clocks in human red blood cells. Nature.

[39] Shi T, Ilikchyan I, Rabouille S, Zehr JP (2010). Genome-wide analysis of diel gene expression in the unicellular N 2-fixing cyanobacterium *Crocosphaera watsonii* WH 8501. ISME J..

[40] Červený J, Sinetova MA, Valledor L, Sherman LA, Nedbal L (2013). Ultradian metabolic rhythm in the diazotrophic cyanobacterium Cyanothece sp. ATCC 51142. Proc. Natl. Acad. Sci. U. S. A..

[41] Johnson CH, Stewart PL, Egli M (2011). The cyanobacterial circadian system: from biophysics to bioevolution. Annu. Rev. Biophys..

[42] Mitsui A (1986). Strategy by which nitrogen-fixing unicellular cyanobacteria grow photoautotrophically. Nature.

[43] Berman-Frank I (2001). Segregation of nitrogen fixation and oxygenic photosynthesis in the marine cyanobacterium *Trichodesmium*. Science.

[44] Besharova O, Suchanek VM, Hartmann R, Drescher K, Sourjik V (2016). Diversification of gene expression during formation of static submerged biofilms by *Escherichia coli*. Front. Microbiol..

[45] Nishiyama Y (2001). Oxidative stress inhibits the repair of photodamage to the photosynthetic machinery. EMBO J..

[46] Nishiyama Y, Allakhverdiev SI, Yamamoto H, Hayashi H, Murata N (2004). Singlet oxygen inhibits the repair of photosystem II by suppressing the translation elongation of the D1 protein in Synechocystis sp. PCC 6803. Biochemistry.

[47] Los, D. A. & Zinchenko, V. V. in *Lipids Photosynth. Essent. Regul. Funct.* (eds. Wada, H. & Murata, N.) 329–348 (Springer Netherlands, 2010). doi:10.1007/978–90–481–2863–1_15

[48] Zehr JP, Crumbliss LL, Church MJ, Omoregie EO, Jenkins BD (2003). Nitrogenase genes in PCR and RT-PCR reagents: implications for studies of diversity of functional genes. Biotechniques.

[49] Stal LJ, Bolhuis H, Cretoiu MS (2019). Phototrophic marine benthic microbiomes: the ecophysiology of these biological entities. Environ. Microbiol..

[50] Bolhuis, H., Severin, I., Confurius-Guns, V., Wollenzien, U. I. a & Stal, L. J. Horizontal transfer of the nitrogen fixation gene cluster in the cyanobacterium *Microcoleus chthonoplastes*. *ISME J.* 4**,** 121–30 (2010). 10.1038/ismej.2009.99 19741736

[51] Lee SH, Pulakat L, Parker KC, Gavini N (1998). Genetic analysis on the NifW by utilizing the yeast two-hybrid system revealed that the NifW of *Azotobacter vinelandii* interacts with the NifZ to form higher-order complexes. Biochem. Biophys. Res. Commun..

[52] Severin I, Stal LJ (2010). *NifH* expression by five groups of phototrophs compared with nitrogenase activity in coastal microbial mats. FEMS Microbiol. Ecol..

[53] Ottesen, E. a *et al.* Pattern and synchrony of gene expression among sympatric marine microbial populations. *Proc. Natl. Acad. Sci. U. S. A.* 110**,** E488–97 (2013). 10.1073/pnas.1222099110 PMC3568374 23345438

[54] Stal LJ, Gemerden H, Krumbein WE (1985). Structure and development of a benthic marine microbial mat. FEMS Microbiol. Lett..

[55] Villbrandt M, Stal LJ (1996). The effect of sulfide on nitrogen fixation in heterocystous and non-heterocystous cyanobacterial mat communities. Algol. Stud. für Hydrobiol. Suppl..

[56] Paerl HW, Pinckney JL, Steppe TF (2000). Cyanobacterial-bacterial mat consortia: Examining the functional unit of microbial survival and growth in extreme environments. Environ. Microbiol..

[57] Fourçans A (2004). Characterization of functional bacterial groups in a hypersaline microbial mat community (Salins-de-Giraud, Camargue, France). FEMS Microbiol. Ecol..

[58] Kearse M (2012). Geneious Basic: an integrated and extendable desktop software platform for the organization and analysis of sequence data. Bioinformatics.

[59] Pabinger S, Rödiger S, Kriegner A, Vierlinger K, Weinhäusel A (2014). A survey of tools for the analysis of quantitative PCR (qPCR) data. Biomol. Detect. Quantif..

