# Supplementary material for: Circadian clock-controlled gene expression in co-cultured, mat-forming cyanobacteria
Source: Sci Rep. 2020 Aug 24;10:14095. doi: 10.1038/s41598-020-69294-3 (PMC7445270; doi:10.1038/s41598-020-69294-3)
Supplement: Supplementary file 1 — Supplementary information [file 41598_2020_69294_MOESM1_ESM.pdf]

Circadian clock-controlled gene expression in co-cultured, mat-forming cyanobacteria.

Christine Hörnlein[a], Veronique Confurius-Guns[a], Michelle Grego[a], Lucas J. Stal[a],[b], Henk Bolhuis[a]

[a] Department of Marine Microbiology and Biogeochemistry, Royal Netherlands Institute for Sea Research, and Utrecht University, Den Burg, Netherlands

[b] Department of Fresh Water and Marine Ecology, Institute for Biodiversity and Ecosystem Dynamics, University of Amsterdam, Amsterdam, Netherlands

Supplementary figure S1A. Direct comparison of average gene expression levels in *L. aestuarii* grown under different conditions. ML = Monoculture-Liquid, CL = Coculture-Liquid, MB = Monoculture-Biofilm, CB = Coculture-Biofilm, DL = grown under dark /light illumination LL= grown under continuous illumination

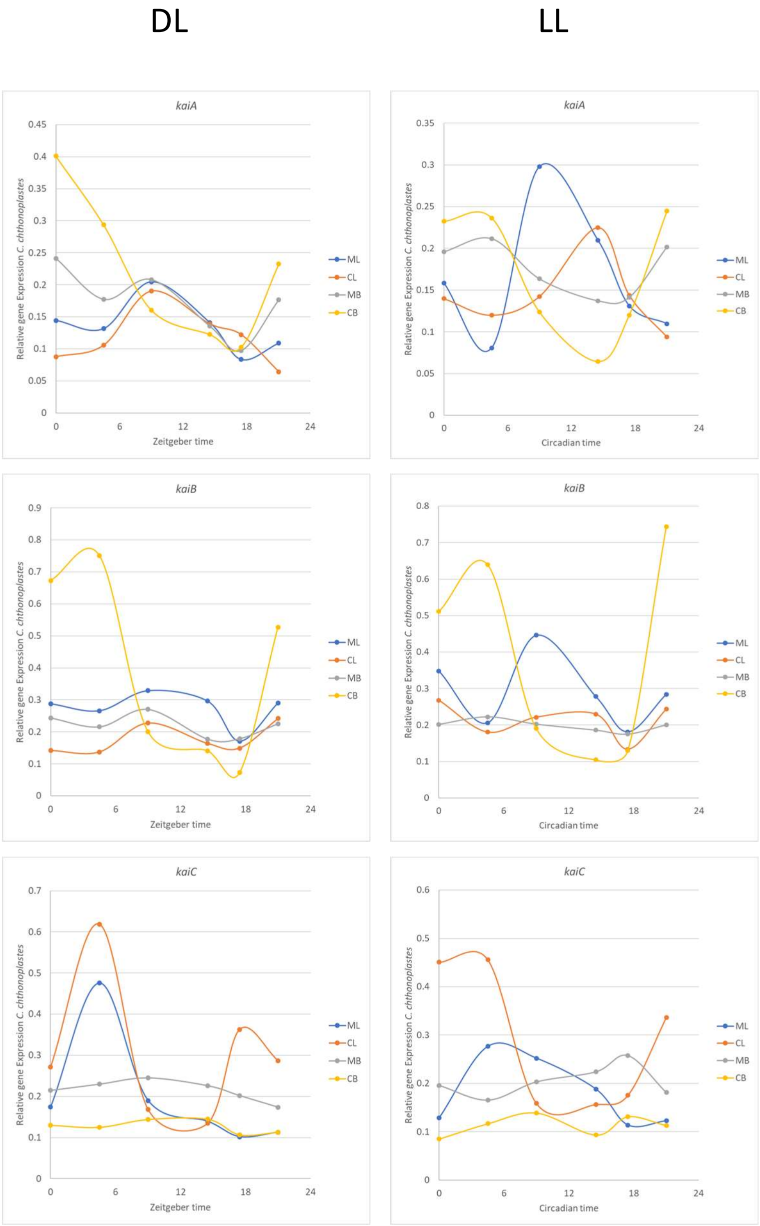

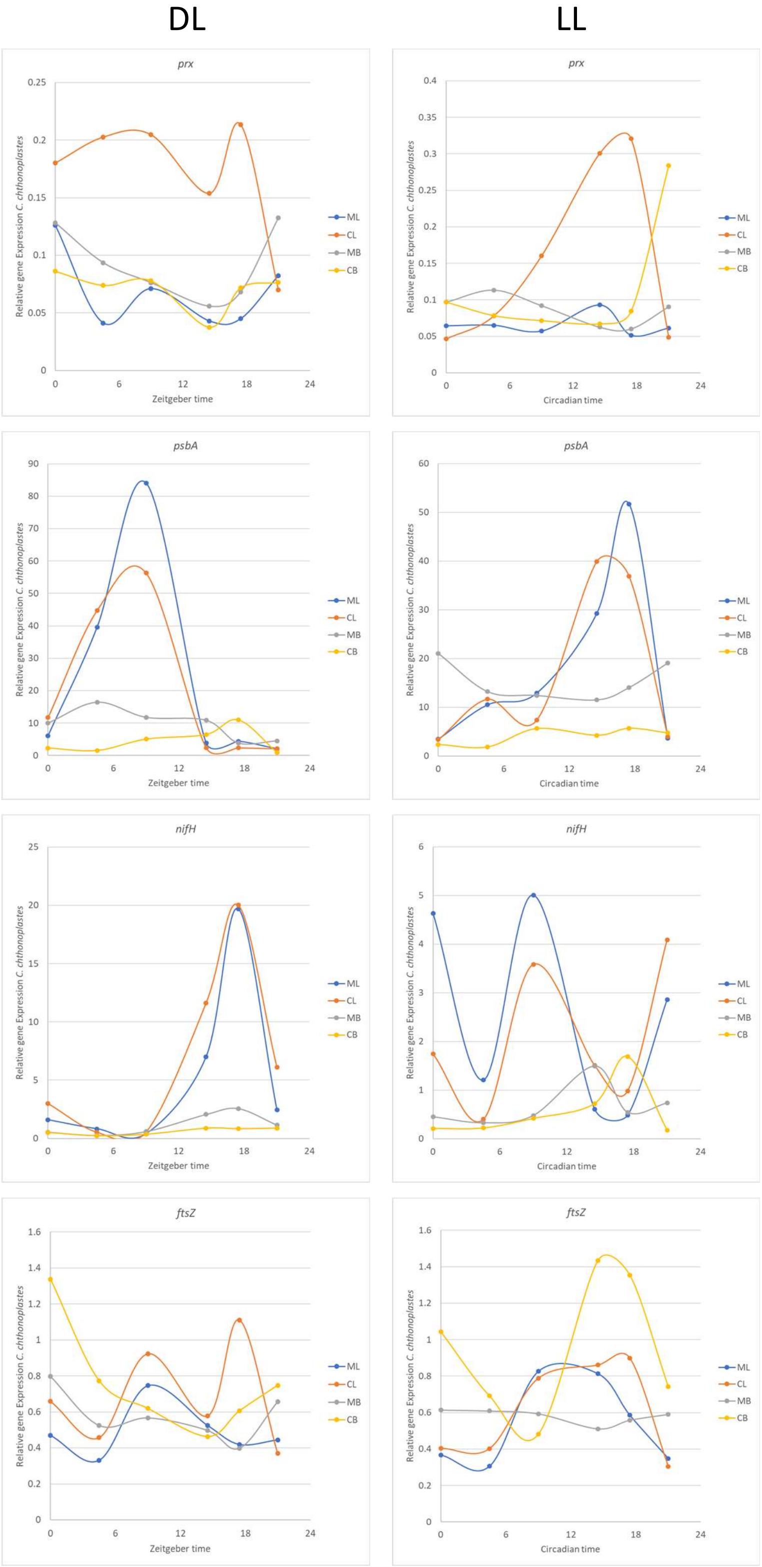

Supplementary figure S1B. Direct comparison of average gene expression levels in *C. chthonoplastes* grown under different conditions. ML = Monoculture-Liquid, CL = Coculture-Liquid, MB = Monoculture-Biofilm, CB = Coculture-Biofilm. DL = grown under dark /light illumination LL= grown under continuous illumination

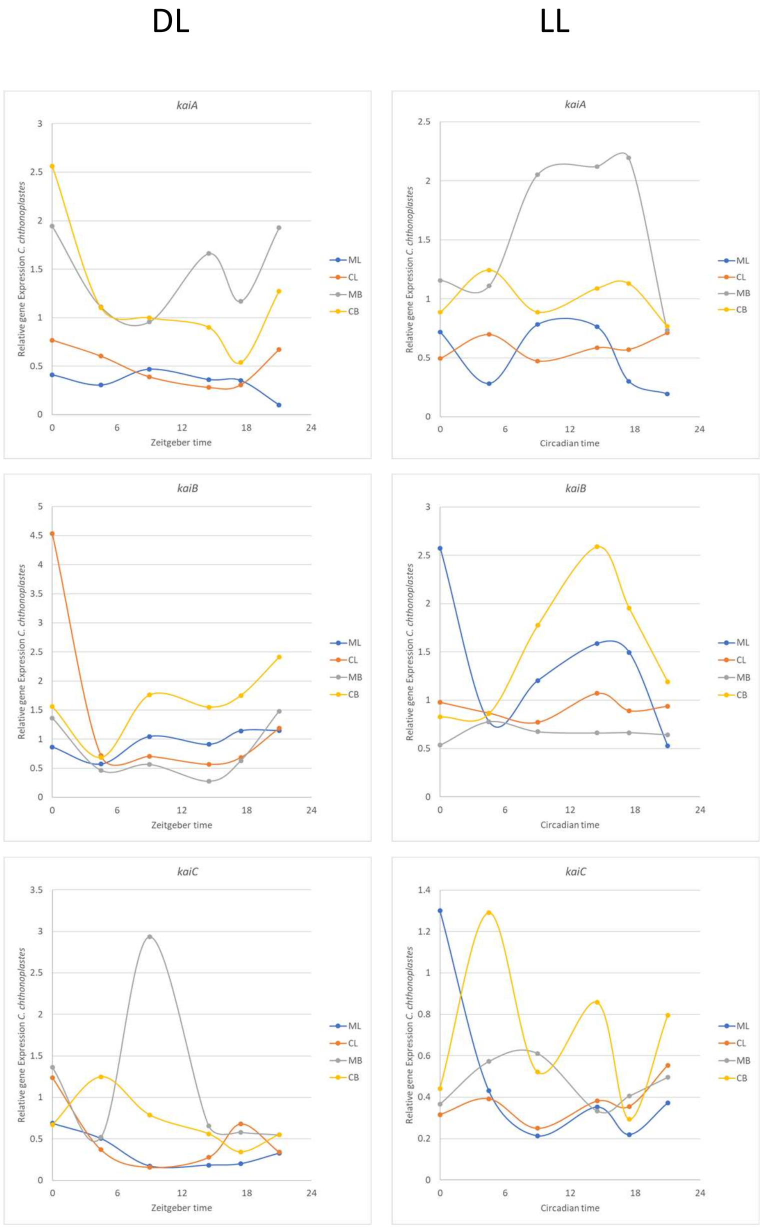

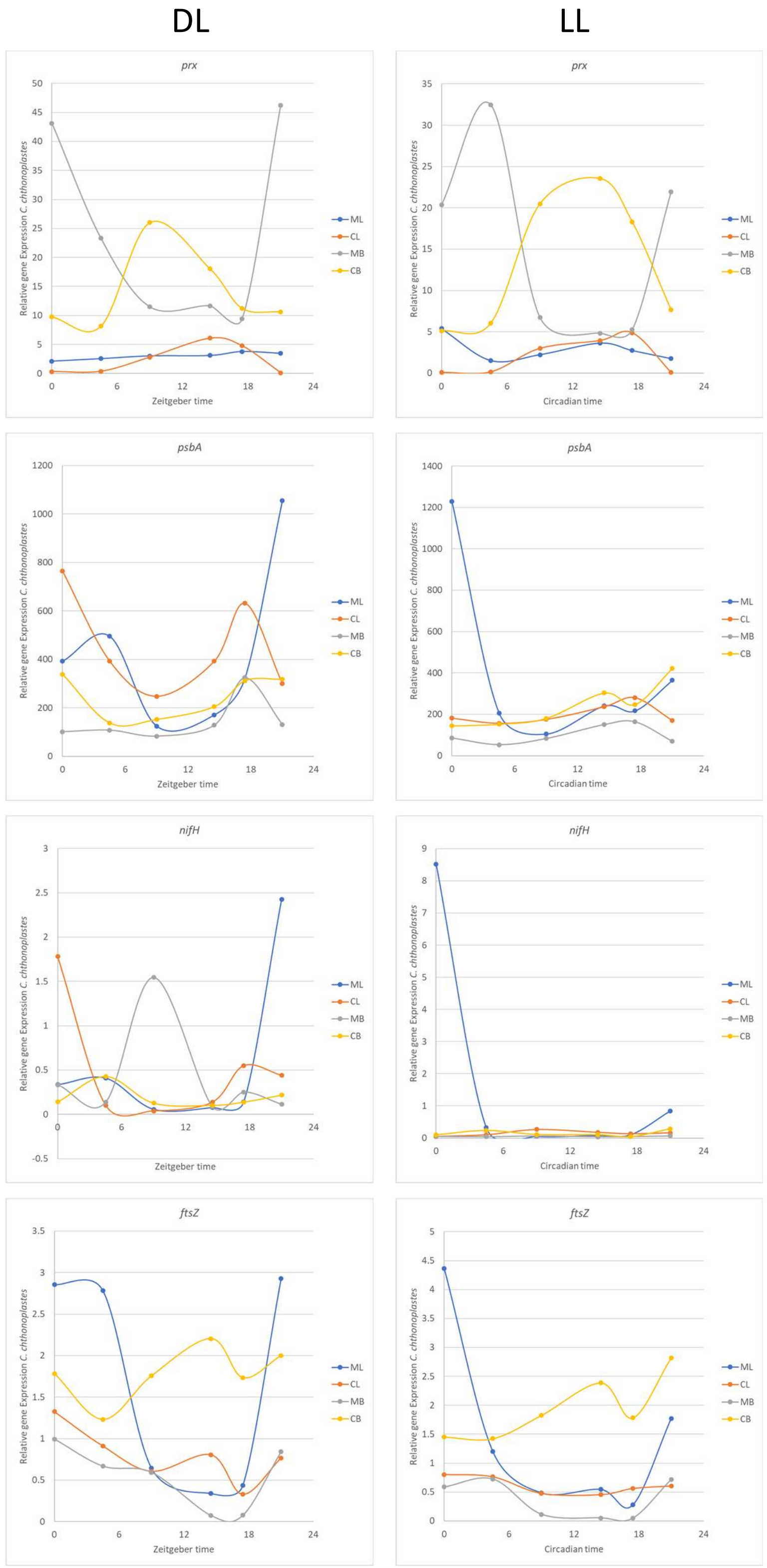

Supplementary Figure S2. Red box indicate significant sinusoidal gene expression patterns as determined by MetaCycle, yellow boxes indicate instances of sunisoidal gene expression under both LD and LL conditions.

Coleofasciculus chthonoplastes LD

	ML	CL	MB	CB
kaiA		x		
kaiB			x	
kaiC	x			x
prx		x	x	x
psbA				x
nifH				
ftsZ	x		x	

Coleofasciculus chthonoplastes LL

	ML	CL	MB	CB
kaiA			x	
kaiB				x
kaiC				
prx		x	x	x
psbA		x	x	
nifH				
ftsZ	x	x	x	

Coleofasciculus chthonoplastes LD&LL

	ML	CL	MB	CB
kaiA				
kaiB				
kaiC				
prx				
psbA		Z	Z	Z
nifH				
ftsZ	Z		Z	

LD = Light/Dark illumination
LL = continuous illumination
ML = Monoculture-Liquid
CL = Coculture-Liquid
MB = Monoculture-Biofilm
CB = Coculture-Biofilm.

Lyngbya aestuarii LD

	ML	CL	MB	CB
kaiA		x		x
kaiB				x
kaiC			x	
prx			x	
psbA	x	x	x	
nifH		x	x	x
ftsZ				x

Lyngbya aestuarii LL

	ML	CL	MB	CB
kaiA			x	x
kaiB			x	x
kaiC	x	x	x	
prx		x	x	
psbA		x	x	
nifH				
ftsZ	x	x	x	

Lyngbya aestuarii LD&LL

	ML	CL	MB	CB
kaiA				Z
kaiB				Z
kaiC			Z	
prx			Z	
psbA		Z	Z	
nifH				
ftsZ				

Significant sinusoidal patterns in *Coleofasciculus chthonoplastes* under LD conditions

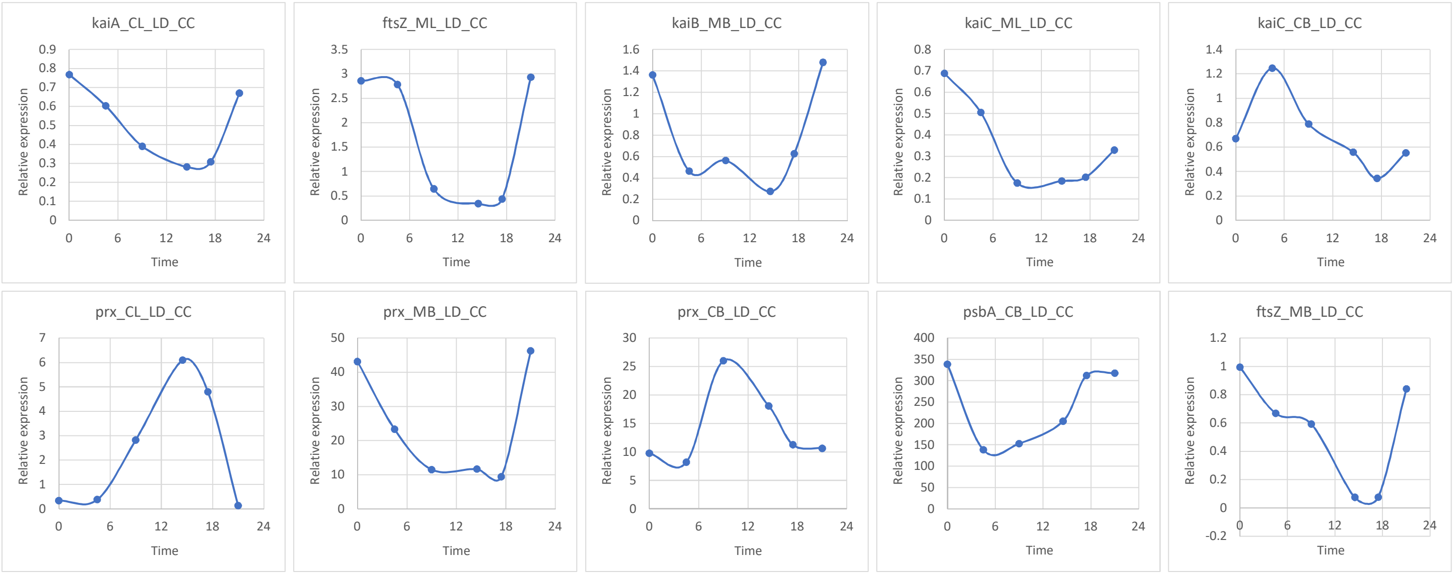

Significant sinusoidal patterns in *Coleofasciculus chthonoplastes* under LL conditions

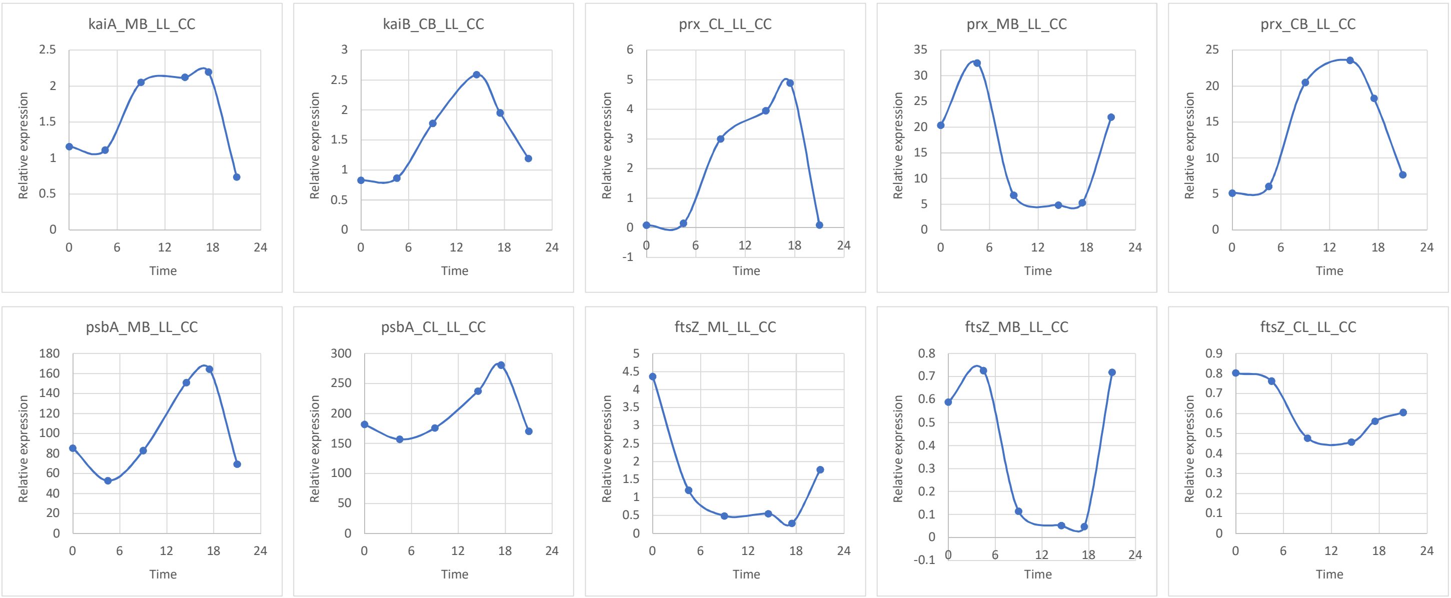

Significant sinusoidal patterns in *Lyngbya aestuarii* under LD conditions

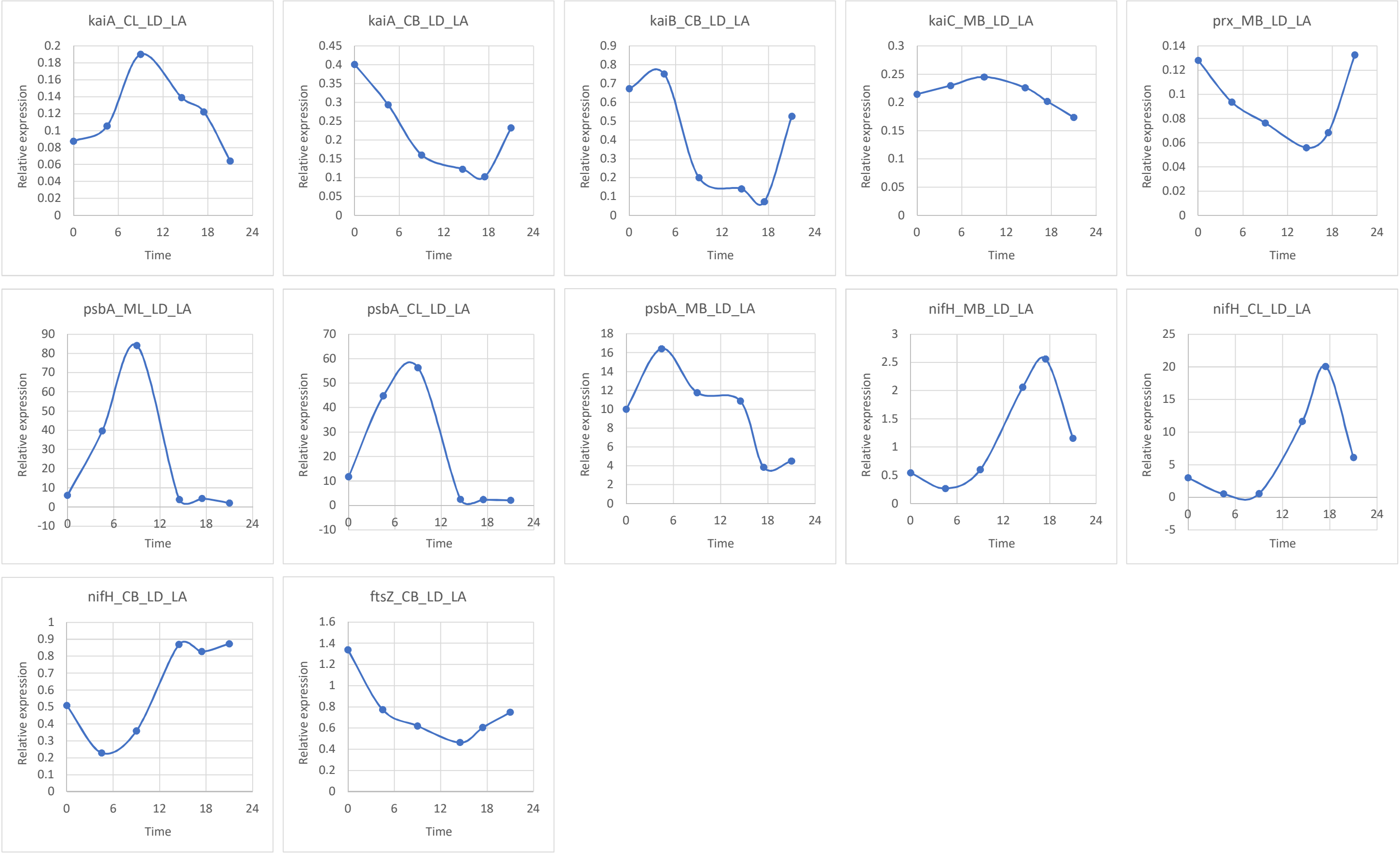

Significant sinusoidal patterns in *Lyngbya aestuarii* under LL conditions

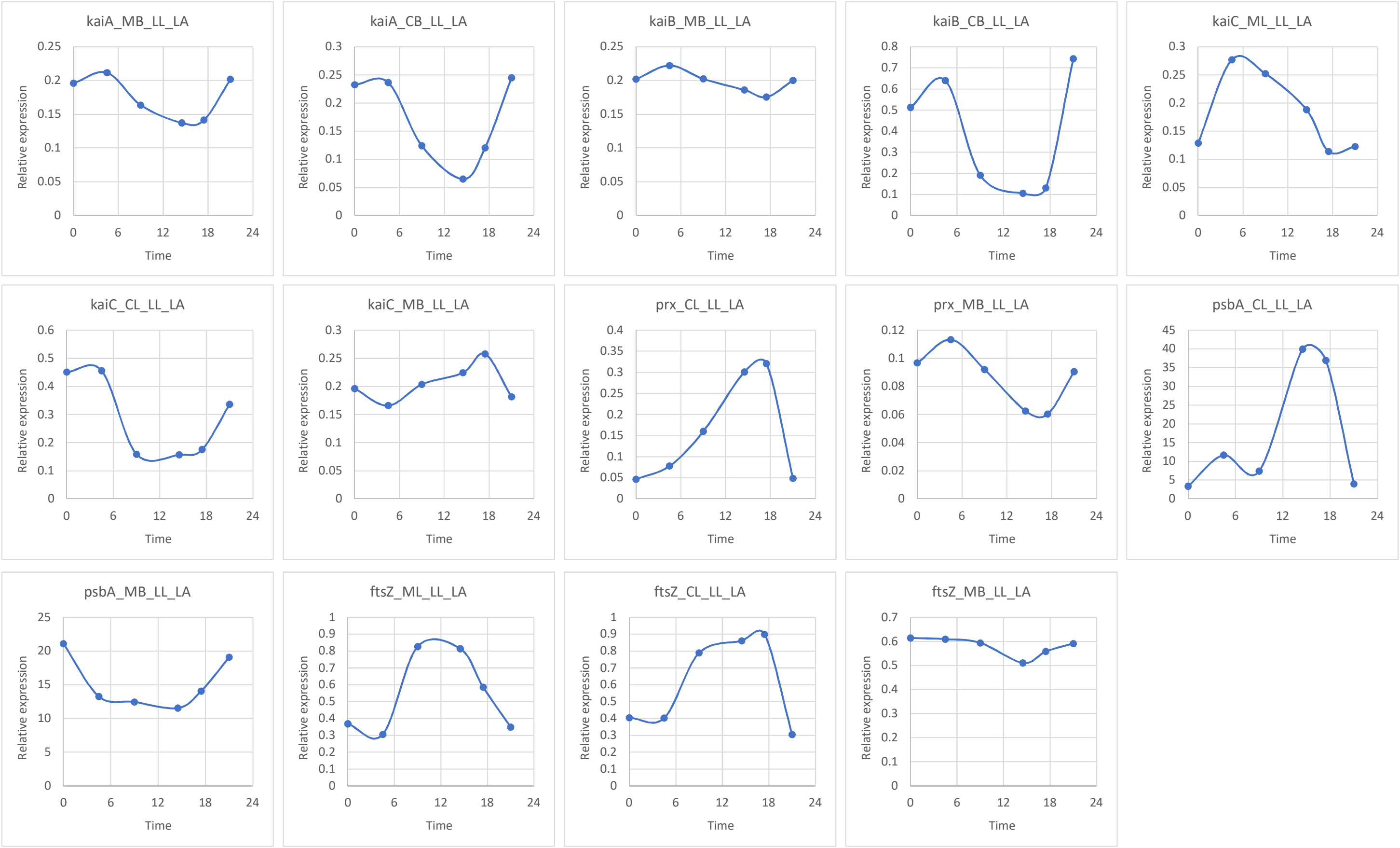

Figure S3 Neighbour-joining nucleotide alignment (ClustalW, 65% identity) and protein alignment of the circadian genes *kaiA*, *kaiB* and *kaiC* (genes in a cluster) between *L. aestuarii* CCY9616 and *C. chthonoplastes* CCY9604 and between *S. elongatus* PCC7941 and *L aestuarii* and *S. elongatus* and *C. chthonoplastes*. Black=identical, grey=similar (65%), white=not similar.

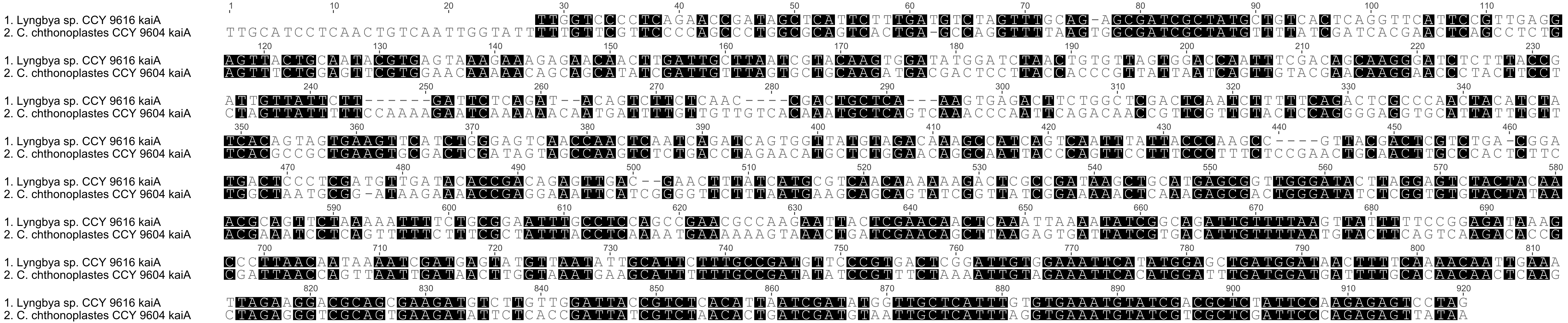
1. Lyngbya sp. CCY 9616 kaiA
2. C. chthonoplastes CCY 9604 kaiA
1
10
20
30
40
50
60
70
80
90
100
110
120
130
140
150
160
170
180
190
200
210
220
230
240
250
260
270
280
290
300
310
320
330
340
350
360
370
380
390
400
410
420
430
440
450
460
470
480
490
500
510
520
530
540
550
560
570
580
590
600
610
620
630
640
650
660
670
680
690
700
710
720
730
740
750
760
770
780
790
800
810
820
830
840
850
860
870
880
890
900
910
920

```
                                                   1        10        20        30        40        50        60        70        80        90       100       110
1. Lyngbya sp. CCY 9616 KaiA translation                   MVPSEPIAHSLMSSLQSDRYAVTQVHSVEELLQYVSKEREQLDCLIVQVDMDLTVLVDQFRQQGISLPIVILDSQIQSS----QPTAQSETSGSTQSFSD-S
2. C. chthonoplastes CCY 9604 KaiA translation    MHPQLSIGIFVRSPALAQSLSQVLSGDRYVLSITNSASEFLEFVEQKQQHIDCLVLQDDDSLPPVINQLYEQGTLLPLVIFPKESKNNDFVVVTNAQSNPIQTTVRCTPGE

                                                      120       130       140       150       160       170       180       190       200       210       220
1. Lyngbya sp. CCY 9616 KaiA translation          PNYIYHSSEVHLGVNQLNQISGYVDKAISQFITQAVTTRLTDDSLDVDTPTELTN--FIMRQQKRLADKLHERLGYLGVYYKRSSKNFLRNLPPAERQELLEQLKLKYRQI
2. C. chthonoplastes CCY 9604 KaiA translation    VHYLFHAAEVRLDSSQVSDLEHALEQAITQFLSLSPNCNLPTLLANADKKTEEIHRGSLMKQQYRLSEKLKERLGYLGVYYKRNPQFFFRYLPQNEKSKLIEQLKSDYRDI

                                                          230       240       250       260       270       280       290       300  305
1. Lyngbya sp. CCY 9616 KaiA translation          VLSYFSGDKALNNKIDEYVNIAFFADVPVTRIVEIHMELMDNFSKQLKLEGRSEDVLLDYRLTLIDMVAHLCEMYRRSIPRES
2. C. chthonoplastes CCY 9604 KaiA translation    VLMYFSQDTAINQLIDNLVNEAFFADISVSKIVEIHMDLMDDFAQQLKLEGRSEDILTDYRLTLIDVIAHLGEMYRRSIPREL
```

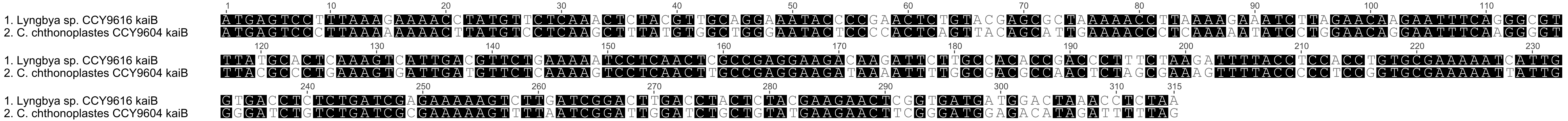
1 10 20 30 40 50 60 70 80 90 100 110
1. Lyngbya sp. CCY9616 kaiB ATGAGTCCTTTAAAGAAAACCTATGTTCTCAAACTCTACGTTGCAGGAAATACCCCGAACTCTGTACGAGCGCTAAAAACCTTAAAAGAAATCTTAGAACAAGAATTTCAGGGCGT
2. C. chthonoplastes CCY9604 kaiB ATGAGTCCCTTAAAAAAAACTTATGTCCTCAAGCTTTATGTGGCTGGGAATACTCCCCACTCAGTTACAGCATTGAAAACCCTCAAAAATATCCTGGAACAGGAATTTCAAGGGGT
120 130 140 150 160 170 180 190 200 210 220 230
1. Lyngbya sp. CCY9616 kaiB TTATGCACTCAAAGTCATTGACGTTCTGAAAAATCCTCAACTCGCCGAGGAAGACAAGATTCTTGCCACACCGACCCTTTCTAAGATTTTACCTCCACCTGTGCGAAAAATCATTG
2. C. chthonoplastes CCY9604 kaiB TTACGCCCTGAAAGTGATTGATGTTCTCAAAAGTCCTCAACTTGCCGAGGAAGATAAAATTTTGGCGACGCCAACTCTAGCGAAAGTTTTACCCCCTCCGGTGCGAAAAATTATTG
240 250 260 270 280 290 300 310 315
1. Lyngbya sp. CCY9616 kaiB GTGACCTCTCTGATCGAGAAAAAGTCTTGATCGGACTTGACCTACTCTACGAAGAACTCGGTGATGATGGACTAAACCTCTAA
2. C. chthonoplastes CCY9604 kaiB GGGATCTGTCTGATCGCGAAAAAGTTTTAATCGGATTGGATCTGCTGTATGAAGAACTTCGGGATGGAGACATAGATTTTTAG

	1	10	20	30	40	50	60	70	80	90	100	104

```
                                                   1        10        20        30        40        50        60        70        80        90       100  104
1. Lyngbya sp. CCY9616 KaiB translation            MSPLKKTYVLKLYVAGNTPNSVRALKTLKEILEQEFQGVYALKVIDVLKNPQLAEEDKILATPTLSKILPPPVRKIIGDLSDREKVLIGLDLLYEELGDDGLNL
2. C. chthonoplastes CCY9604 KaiB translation      MSPLKKTYVLKLYVAGNTPHSVTALKTLKNILEQEFQGVYALKVIDVLKSPQLAEEDKILATPTLAKVLPPPVRKIIGDLSDREKVLIGLDLLYEELRDGDIDF
```

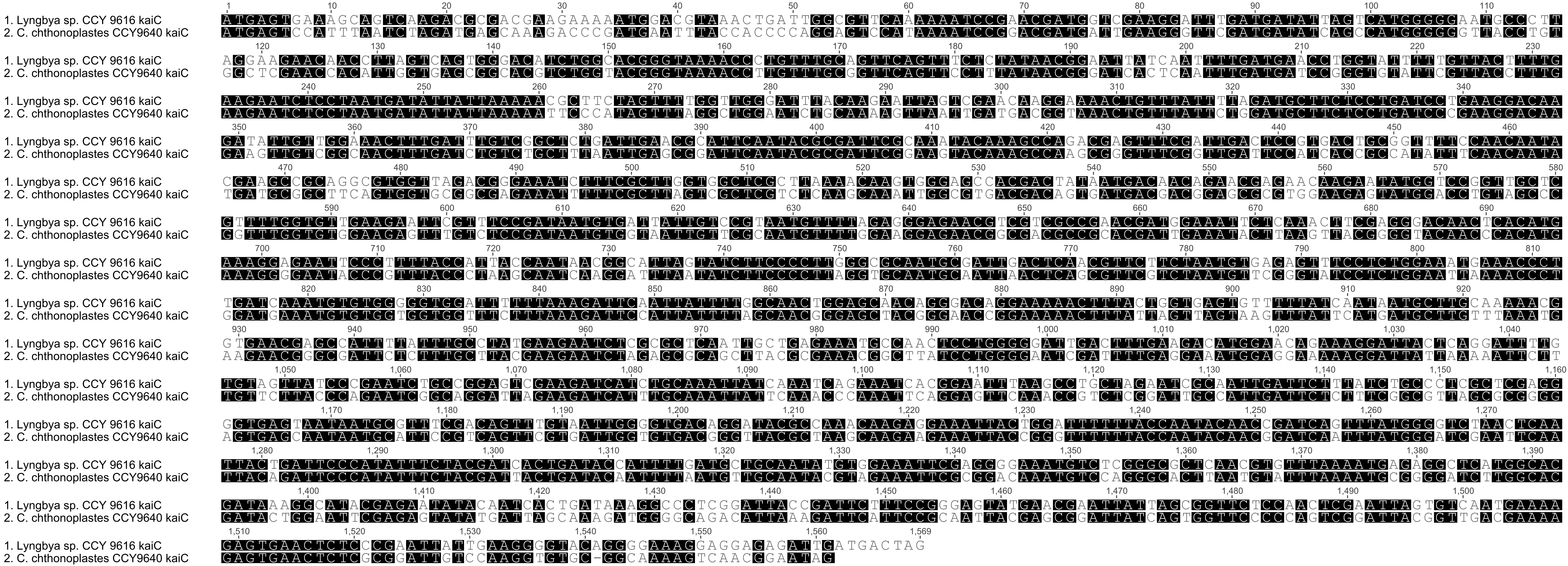
1. Lyngbya sp. CCY 9616 kaiC
2. C. chthonoplastes CCY9640 kaiC

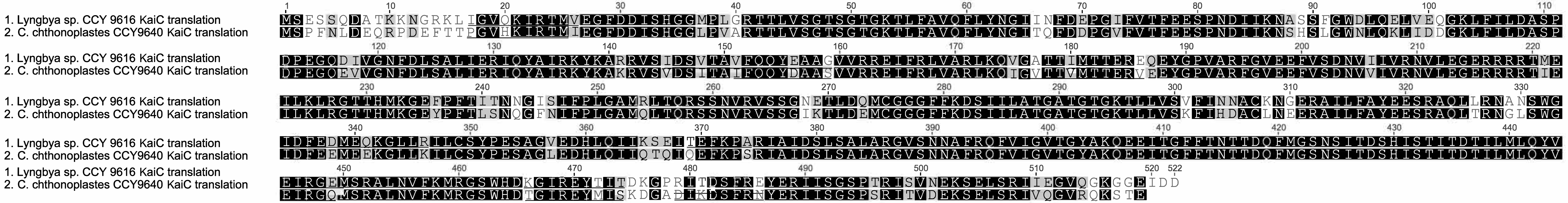
1. Lyngbya sp. CCY 9616 KaiC translation MSESSQDATKKNGRKLIGVQKIRTMVEGFDDISHGGMPLGRTTLVSGTSGTGKTLFAVQFLYNGIINFDEPGIFVTFEESPNDIIKNASSFGWDLQELVEQGKLFILDASP
2. C. chthonoplastes CCY9640 KaiC translation MSPFNLDEQRPDEFTTPGVHKIRTMIEGFDDISHGGLPVARTTLVSGTSGTGKTLFAVQFLYNGITQFDDPGVFVTFEESPNDIIKNSHSLGWNLQKLIDDGKLFILDASP
1. Lyngbya sp. CCY 9616 KaiC translation DPEGQDIVGNFDLSALIERIQYAIRKYKARRVSIDSVTAVFQQYEAAGVVRREIFRLVARLKQVGATTIMTTEREQEYGPVARFGVEEFVSDNVIIVRNVLEGERRRRTME
2. C. chthonoplastes CCY9640 KaiC translation DPEGQEVVGNFDLSALIERIQYAIRKYKAKRVSVDSITAIFQQYDAASVVRREIFRLVARLKQIGVTTVMTTERVEEYGPVARFGVEEFVSDNVVIVRNVLEGERRRRTIE
1. Lyngbya sp. CCY 9616 KaiC translation ILKLRGTTHMKGEFPFTITNNGISIFPLGAMRLTQRSSNVRVSSGNETLDQMCGGGFFKDSIILATGATGTGKTLLVSVFINNACKNGERAILFAYEESRAQLLRNANSWG
2. C. chthonoplastes CCY9640 KaiC translation ILKLRGTTHMKGEYPFTLSNQGFNIFPLGAMQLTQRSSNVRVSSGIKTLDEMCGGGFFKDSIILATGATGTGKTLLVSKFIHDACLNEERAILFAYEESRAQLTRNGLSWG
1. Lyngbya sp. CCY 9616 KaiC translation IDFEDMEQKGLLRILCSYPESAGVEDHLQIIKSEITEFKPARIAIDSLSALARGVSNNAFRQFVIGVTGYAKQEEITGFFTNTTDQFMGSNSITDSHISTITDTILMLQYV
2. C. chthonoplastes CCY9640 KaiC translation IDFEEMEEKGLLKILCSYPESAGLEDHLQIIQTQIQEFKPSRIAIDSLSALARGVSNNAFRQFVIGVTGYAKQEEITGFFTNTTDQFMGSNSITDSHISTITDTILMLQYV
1. Lyngbya sp. CCY 9616 KaiC translation EIRGEMSRALNVFKMRGSWHDKGIREYTITDKGPRITDSFREYERIISGSPTRISVNEKSELSRIIEGVQGKGGEIDD
2. C. chthonoplastes CCY9640 KaiC translation EIRGQMSRALNVFKMRGSWHDTGIREYMISKDGADIKDSFRNYERIISGSPSRITVDEKSELSRIVQGVRQKSTE

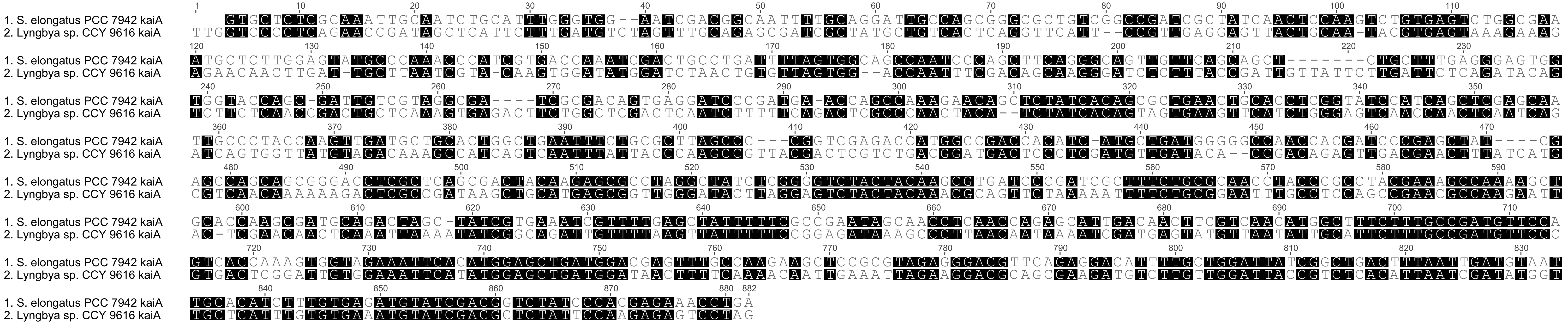
1. S. elongatus PCC 7942 kaiA
2. Lyngbya sp. CCY 9616 kaiA

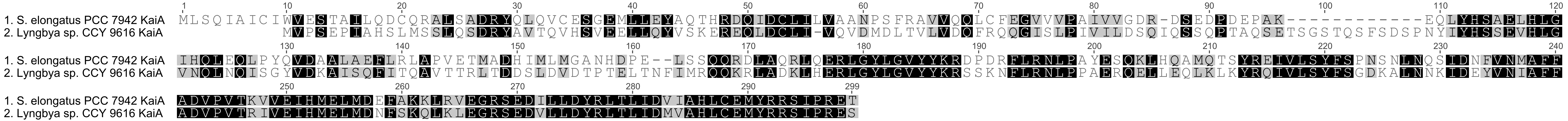
1. S. elongatus PCC 7942 KaiA
MLSQIAICIWVESTAILQDCQRALSADRYQLQVCESGEMLLEYAQTHRDQIDCLILVAANPSFRAVVQQLCFEGVVVPAIVVGDR-DSEDPDEPAK------------EQLYHSAELHLG
2. Lyngbya sp. CCY 9616 KaiA
MVPSEPIAHSLMSSLQSDRYAVTQVHSVEELLQYVSKEREQLDCLI-VQVDMDLTVLVDQFRQQGISLPIVILDSQIQSSQPTAQSETSGSTQSFSDSPNYIYHSSEVHLG
1. S. elongatus PCC 7942 KaiA
IHQLEQLPYQVDAALAEFLRLAPVETMADHIMLMGANHDPE--LSSQQRDLAQRLQERLGYLGVYYKRDPDRFLRNLPAYESQKLHQAMQTSYREIVLSYFSPNSNLNQSIDNFVNMAFF
2. Lyngbya sp. CCY 9616 KaiA
VNQLNQISGYVDKAISQFITQAVTTRLTDDSLDVDTPTELTNFIMRQQKRLADKLHERLGYLGVYYKRSSKNFLRNLPPAERQELLEQLKLKYRQIVLSYFSGDKALNNKIDEYVNIAFF
1. S. elongatus PCC 7942 KaiA
ADVPVTKVVEIHMELMDEFAKKLRVEGRSEDILLDYRLTLIDVIAHLCEMYRRSIPRET
2. Lyngbya sp. CCY 9616 KaiA
ADVPVTRIVEIHMELMDNFSKQLKLEGRSEDVLLDYRLTLIDMVAHLCEMYRRSIPRES

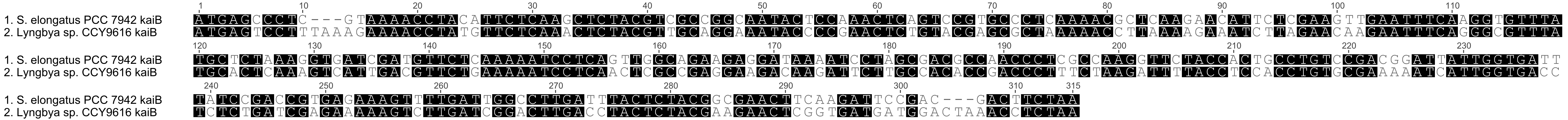
1. S. elongatus PCC 7942 kaiB
2. Lyngbya sp. CCY9616 kaiB

```
                                   1       10        20        30        40        50        60        70        80        90        100  105
1. S. elongatus PCC 7942 KaiB      MSP-RKTYILKLYVAGNTPNSVRALKTLKNILEVEFQGVYALKVIDVLKNPQLAEEDKILATPTLAKVLPLPVRRIIGDLSDREKVLIGLDLLYGELQDSDDF
2. Lyngbya sp. CCY9616 KaiB        MSPLKKTYVLKLYVAGNTPNSVRALKTLKEILEQEFQGVYALKVIDVLKNPQLAEEDKILATPTLSKILPPPVRKIIGDLSDREKVLIGLDLLYEELGD-DGLNL
```

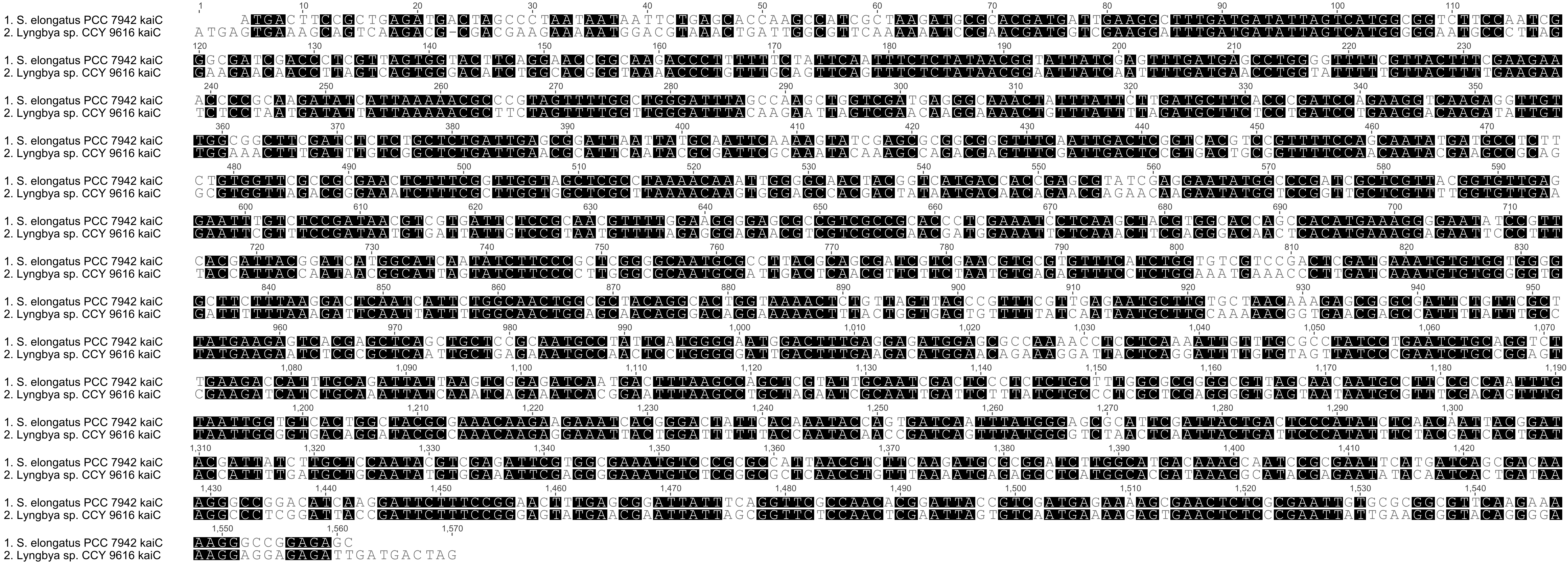
1. S. elongatus PCC 7942 kaiC
2. Lyngbya sp. CCY 9616 kaiC

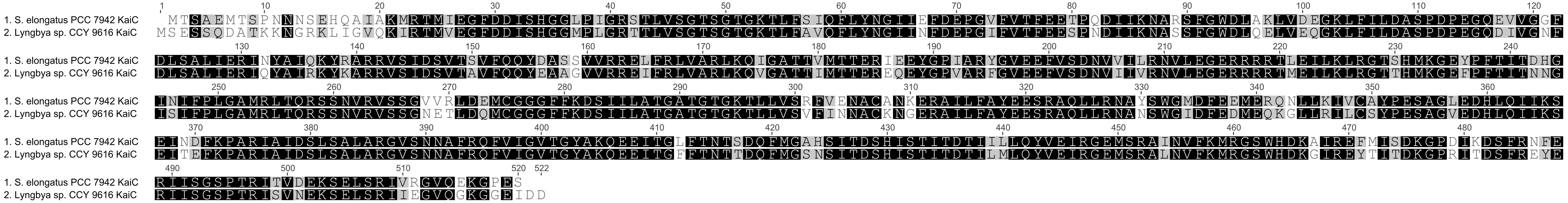
1. S. elongatus PCC 7942 KaiC
2. Lyngbya sp. CCY 9616 KaiC

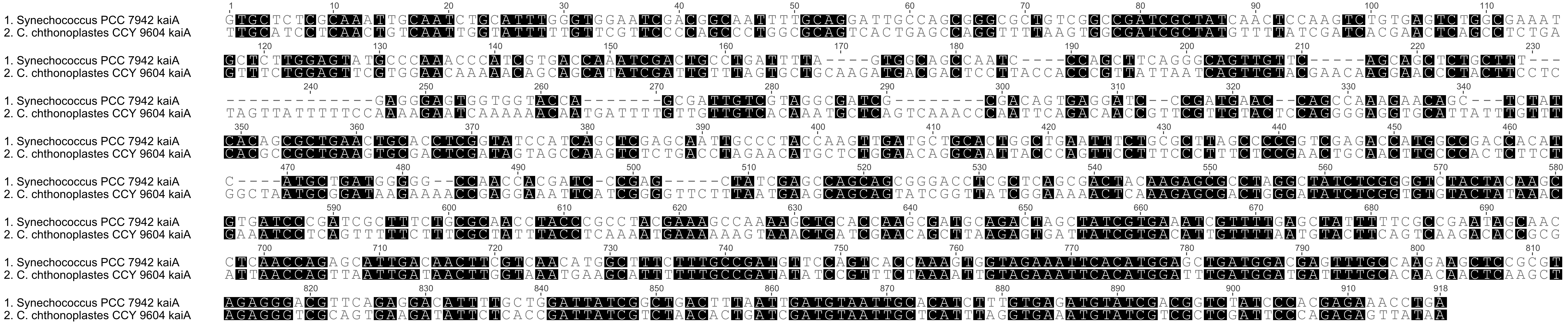
1. Synechococcus PCC 7942 kaiA
2. C. chthonoplastes CCY 9604 kaiA

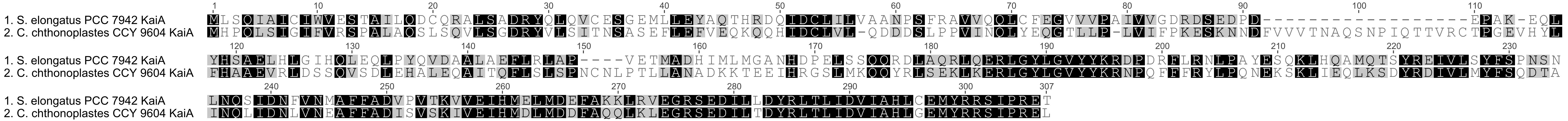

1 10 20 30 40 50 60 70 80 90 100 110
1. S. elongatus PCC 7942 KaiA MLSQIAICIWVESTAILQDCQRALSADRYQLQVCESGEMLLEYAQTHRDQIDCLILVAANPSFRAVVQQLCFEGVVVPAIVVGDRDSEDPD------------------EPAK-EQL
2. C. chthonoplastes CCY 9604 KaiA MHPQLSIGIFVRSPALAQSLSQVLSGDRYVLSITNSASEFLEFVEQKQQHIDCLVL-QDDDSLPPVINQLYEQGTLLP-LVIFPKESKNNDFVVVTNAQSNPIQTTVRCTPGEVHYL
120 130 140 150 160 170 180 190 200 210 220 230
1. S. elongatus PCC 7942 KaiA YHSAELHLGIHQLEQLPYQVDAALAEFLRLAP----VETMADHIMLMGANHDPELSSQQRDLAQRLQERLGYLGVYYKRDPDRFLRNLPAYESQKLHQAMQTSYREIVLSYFSPNSN
2. C. chthonoplastes CCY 9604 KaiA FHAAEVRLDSSQVSDLEHALEQAITQFLSLSPNCNLPTLLANADKKTEEIHRGSLMKQQYRLSEKLKERLGYLGVYYKRNPQFFRYLPQNEKSKLIEQLKSDYRDIVLMYFSQDTA
240 250 260 270 280 290 300 307
1. S. elongatus PCC 7942 KaiA LNQSIDNFVNMAFFADVPVTKVVEIHMELMDEFAKKLRVEGRSEDILLDYRLTLIDVIAHLCEMYRRSIPRET
2. C. chthonoplastes CCY 9604 KaiA INQLIDNLVNEAFFADISVSKIVEIHMDLMDDFAQQLKLEGRSEDILTDYRLTLIDVIAHLGEMYRRSIPREL

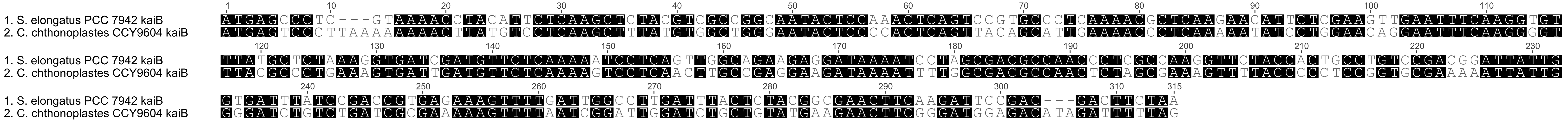
1. S. elongatus PCC 7942 kaiB
2. C. chthonoplastes CCY9604 kaiB

```
                                1        10        20        30        40        50        60        70        80        90       100 104
1. S. elongatus PCC 7942 KaiB   MSP-RKTYILKLYVAGNTPNSVRALKTLKNILEVEFQGVYALKVIDVLKNPQLAEEDKILATPTLAKVLPLPVRRIIGDLSDREKVLIGLDLLYGELQDSD-DF
2. C. chthonoplastes CCY9604 KaiB MSPLKKTYVLKLYVAGNTPHSVTALKTLKNILEQEFQGVYALKVIDVLKSPQLAEEDKILATPTLAKVLPPPVRKIIGDLSDREKVLIGLDLLYEELRDGDIDF
```

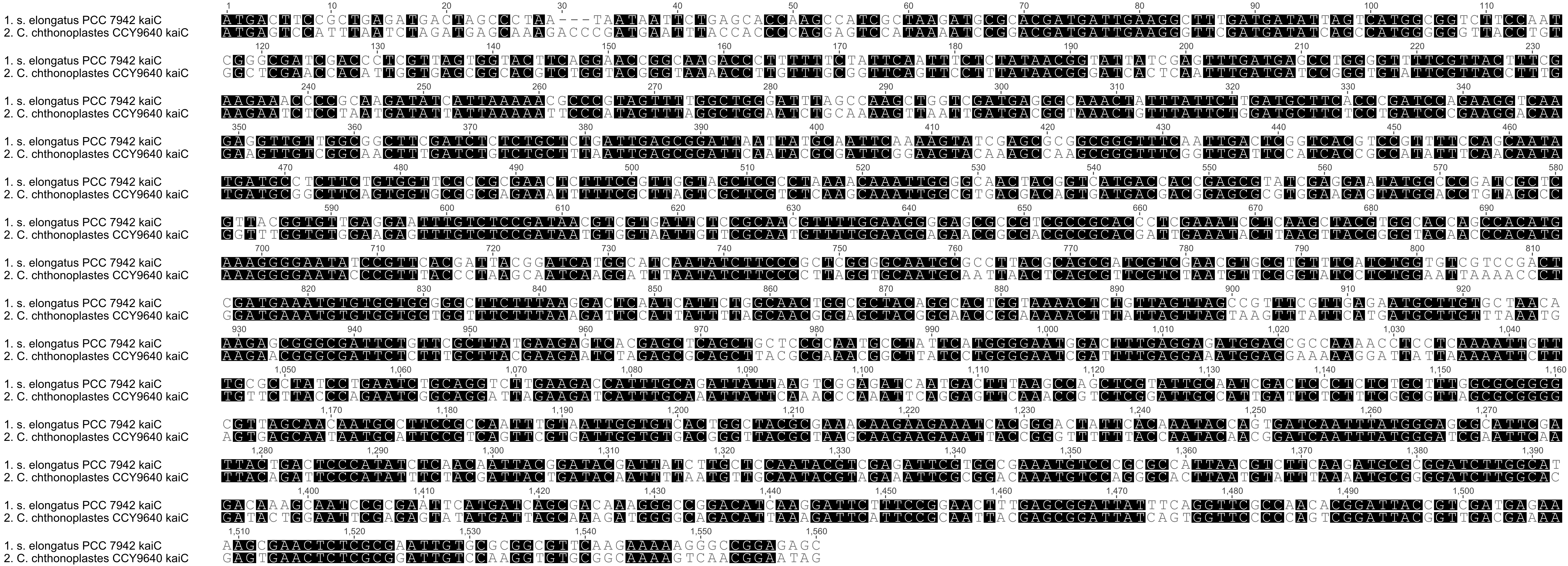
1. s. elongatus PCC 7942 kaiC
2. C. chthonoplastes CCY9640 kaiC

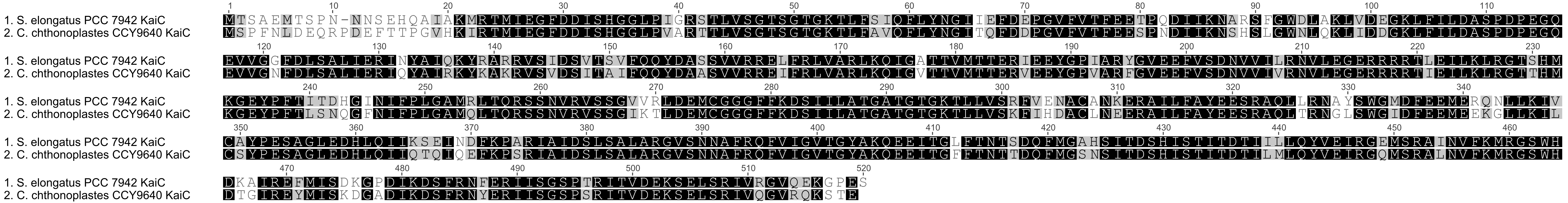
1. S. elongatus PCC 7942 KaiC
2. C. chthonoplastes CCY9640 KaiC
MTSAEMTSPN-NNSEHQAIAKMRTMIEGFDDISHGGLPIGRSTLVSGTSGTGKTLFSIQFLYNGIIEFDEPGVFVTFEETPQDIIKNARSFGWDLAKLVDEGKLFILDASPDPEGQ
MSPFNLDEQRPDEFTTPGVHKIRTMIEGFDDISHGGLPVARTTLVSGTSGTGKTLFAVQFLYNGITQFDDPGVFVTFEESPNDIIKNSHSLGWNLQKLIDDGKLFILDASPDPEGQ
EVVGGFDLSALIERINYAIQKYRARRVSIDSVTSVFQQYDASSVVRRELFRLVARLKQIGATTVMTTERIEEYGPIARYGVEEFVSDNVVILRNVLEGERRRRTLEILKLRGTSHM
EVVGNFDLSALIERIQYAIRKYKAKRVSVDSITAIFQQYDAASVVRREIFRLVARLKQIGVTTVMTTERVEEYGPVARFGVEEFVSDNVVIVRNVLEGERRRRTIEILKLRGTTHM
KGEYPFTITDHGINIFPLGAMRLTQRSSNVRVSSGVVRLDEMCGGGFFKDSIILATGATGTGKTLLVSRFVENACANKERAILFAYEESRAQLLRNAYSWGMDFEEMERQNLLKIV
KGEYPFTLSNQGFNIFPLGAMQLTQRSSNVRVSSGIKTLDEMCGGGFFKDSIILATGATGTGKTLLVSKFIHDACLNEERAILFAYEESRAQLTRNGLSWGIDFEEMEKGLLKIL
CAYPESAGLEDHLQIIKSEINDFKPARIAIDSLSALARGVSNNAFRQFVIGVTGYAKQEEITGLFTNTSDQFMGAHSITDSHISTITDTIILLQYVEIRGEMSRAINVFKMRGSWH
CSYPESAGLEDHLQIIQTQIQEFKPSRIAIDSLSALARGVSNNAFRQFVIGVTGYAKQEEITGFFTNTTDQFMGSNSITDSHISTITDTILMLQYVEIRGQMSRALNVFKMRGSWH
DKAIREFMISDKGPDIKDSFRNFERIISGSPTRITVDEKSELSRIVRGVQEKGPES
DTGIREYMISKDGADIKDSFRNYERIISGSPSRITVDEKSELSRIVQGVRQKSTE

Table S1 Alignment of nucleotide and protein sequences of the circadian clock genes *kaiA*, *kaiB* and *kaiC*. Results show the percentage of bases/residues which are identical between *L. aestuarii* CCY 9616 and *C. chthonoplastes* CCY 9604 (A) and when aligned to the circadian clock gene sequences of *S. elongatus* PCC 7942 (B).

A

	C. chthonoplastes					
	**nucleobases**			**protein**		
**Species**	***kaiA***	***kaiB***	***kaiC***	**KaiA**	**KaiB**	**KaiC**
L. aestuarii	56%	75%	75%	46%	88%	82%

B

	S. elongatus					
	**nucleobases**			**protein**		
**Species**	***kaiA***	***kaiB***	***kaiC***	**KaiA**	**KaiB**	**KaiC**
L. aestuarii	54%	70%	71%	46%	86%	81%
C. chthonoplastes	51%	71%	71%	44%	88%	80%

Supplementary Table S2. Minimum and maximum CT values for *kaiABC*, *prx*, *psbA*, *nifH* and *ftsZ* are given per cyanobacterial species and light regime (LD/LL).

Species	Light regime	*kaiA*	*kaiB*	*kaiC*	*prx*	*psbA*	*nifH*	*ftsZ*
L. aestuarii	LD	27 / 34	28 / 35	28 / 35	31 / 37	23 / 33	26 / 35	25 / 32
C. chthonoplastes	LD	27 / 35	28 / 36	26 / 35	30 / 36	22 / 31	26 / 36	24 / 32
L. aestuarii	LL	33 / 38	34 / 38	36 / 38	29 / 36	24 /30	34 / 36	34 / 39
C. chthonoplastes	LL	32 / 37	33 / 37	35 / 38	28 / 34	24 / 28	34 / 37	33 / 39
